# Global dysregulation of circular RNAs in frontal cortex and whole blood from DM1 and DM2

**DOI:** 10.1007/s00439-025-02729-x

**Published:** 2025-02-04

**Authors:** Arvind Srinivasan, Dorota Magner, Piotr Kozłowski, Anna Philips, Arkadiusz Kajdasz, Paweł Wojciechowski, Marzena Wojciechowska

**Affiliations:** 1 Department of Rare Diseases, Institute of Bioorganic Chemistry, Polish Academy of Sciences, Poznan, Poland; 2 Department of Molecular Genetics, Institute of Bioorganic Chemistry, Polish Academy of Sciences, Poznan, Poland; 3 Department of Bioinformatics, Institute of Bioorganic Chemistry, Polish Academy of Sciences, Poznan, Poland; 4 Laboratory of Genomics, Institute of Bioorganic Chemistry, Polish Academy of Sciences, Poznan, Poland; 5 Institute of Computing Science, Poznan University of Technology, Poznan, Poland; 6 Present Address: Department of Biochemistry and Biotechnology, University of Life Sciences, Poznan, Poland

**Keywords:** Circular RNAs, Myotonic dystrophy, Back-splicing, cryptic splice sites, Alternative splicing

## Abstract

Myotonic dystrophy type 1 (DM1) and type 2 (DM2) are autosomal dominant neuromuscular disorders associated with expansions of microsatellites, respectively, in *DMPK* and *CNBP*. Their pathogenesis is linked to the global aberrant alternative splicing (AAS) of many genes and marks mostly muscular and neuronal tissues, while blood is the least affected. Recent data in DM1 skeletal muscles indicated that abnormalities in RNA metabolism also include global upregulation of circular RNAs (circRNAs). CircRNAs are a heterogeneous group considered splicing errors and by-products of canonical splicing. To elucidate whether circRNA dysregulation is an inherent feature of the myotonic environment, we perform their analysis in the frontal cortex and whole blood of DM1 and DM2 patients. We find a global elevation of circRNAs in both tissues, and its magnitude is neither correlated with the differences in their parental gene expression nor is associated with AAS published earlier. Aberrantly spliced cassette exons of linear transcripts affected in DM1 and DM2 are not among the circularized exons, which unique genomic features prerequisite back-splicing. However, the blueprint of the AAS of linear RNAs is found in a variety of circRNA isoforms. The heterogeneity of circRNAs also originates from the utilization of exonic and intronic cryptic donors/acceptors in back splice junctions, and intron-containing circRNAs are more characteristic of the blood. Overall, this study reveals circRNA dysregulation in various tissues from DM1 and DM2; however, their levels do not correlate with the AAS in linear RNAs, suggesting a potential independent regulatory mechanism underlying circRNA upregulation in myotonic dystrophy.

**Supplementary Information:**

The online version contains supplementary material available at 10.1007/s00439-025-02729-x.

## Introduction

Myotonic dystrophy type 1 (DM1) and type 2 (DM2) are rare neuromuscular genetic disorders. Currently, there is no cure or effective treatment to halt or reverse their progression; thus, there is an ongoing search for new biomarkers. DM1 is caused by instability of CTG repeats in the 3’-UTR of *DMPK*, whereas DM2 is associated with CCTG repeat expansion in the first intron of *CNBP* (Brook et al. 1992; Mahadevan et al. 1992; Margolis et al. 2006). The pathogenesis of both disorders is linked to the toxicity of mutation-containing transcripts, which is manifested through their nuclear accumulation in characteristic foci (Wojciechowska M and Krzyzosiak WJ, 2011). These foci attract RNA-binding proteins, including MBNL1, which in turn lead to global changes in alternative splicing (AS). However, the pathogenesis of DM1 and DM2 is also marked by abnormalities in many other RNA metabolism pathways (Wang et al. 2012). Recent results in DM1 have shown a global upregulation of circRNAs in patients’ skeletal muscles and in DM1 transgenic mice (Czubak et al. 2019a, b; Voellenkle et al. 2019).

CircRNAs are derived from both protein-coding and non-coding regions of the genome, exhibiting significant diversity in length and structure. They are formed through a distinct process known as back-splicing, although the precise details of this process remain unclear. However, it is known that back-splicing is influenced by both *cis*-regulatory elements and *trans*-acting RNA-binding proteins (RBPs). CircRNAs can be categorized into three major types: exonic (E-circRNAs), exonic-intronic (E-I circRNAs), and intronic (I-circRNAs) (Ragan et al. 2019; Li et al. 2015; Sunagawa et al. 2021). Several mechanisms have been proposed to explain their production, including the circularization of one or more exons facilitated by repetitive sequences in flanking introns, the use of cryptic splice sites, inefficient debranching of intronic lariats, and the retention of introns in linear transcripts (Ragan et al. 2019; Li et al. 2015; Sunagawa et al. 2021; Hu et al. 2021; Rahimi et al. 2021).

Among the most studied cis-regulatory elements that facilitate back-splicing are the presence of large introns and their short inverted Alu repeats, which flank certain exons and promote circularization through reverse complementary matches (RCM) and/or RNA binding proteins (Jeck et al. 2013; Liang D and Wilusz JE, 2014). However, not all exons flanked by long introns undergo circularization. The efficiency of exon circularization has also been linked to exon size, though this remains a subject of debate. Some studies suggest that longer exons circularize more efficiently, while others propose that smaller exons are more prone to forming circRNAs (Zhang et al. 2014; Szabo et al. 2015). Importantly, many exons that form circRNAs are not part of alternatively spliced cassette exons (CEs), indicating that their unique genomic features, along with the availability of splicing factors, likely influence whether forward or back-splicing occurs (Zhang et al. 2014; Shen et al. 2022; Starke et al. 2015). Among the *trans*-acting RBPs that influence back-splicing are those that either facilitate or inhibit circRNA formation. Muscleblind-like protein (Mbl), the orthologue of human Muscleblind-like proteins MBNL1–3, was the first identified facilitator of exon circularization in *Drosophila* (Ashwal-Fluss et al. 2014). Subsequent studies have identified other RBPs that regulate exon circularization across various systems and organisms (Conn et al. 2015; Ivanov et al. 2015; Aktaş et al. 2017; Zhang et al. 2013; Kramer et al. 2015; Kokot et al. 2022). One proposed mechanism for circRNA biogenesis suggests that RBPs facilitate exon circularization by binding to specific motifs in the flanking introns, thereby promoting intron-intron interactions. The splicing factor MBL/MBNL1 has been shown to control exon circularization in this manner. In both humans and flies, introns flanking the second exon of the MBNL1/Mbl gene, which forms circMBNL1/circMbl, contain highly conserved MBNL-binding motifs. Although downregulation of Mbl in Drosophila cell cultures and neural tissue results in a significant decrease in circMbl levels (Ashwal-Fluss et al. 2014), depletion of MBNL1 in DM1 skeletal muscles leads to elevated levels of circRNAs, as demonstrated by two independent research groups (Czubak et al. 2019a, b; Voellenkle et al. 2019). These findings raise questions about the general role of MBNL1/Mbl in circRNA biogenesis, though they do not exclude its involvement in regulating a subset of circRNA transcripts.

To investigate whether dysregulation of circRNAs is another characteristic of DM1 and DM2 pathogenesis, we implemented a bioinformatics pipeline and re-analyzed publicly available RNA sequencing data from human tissues, specifically the frontal cortex (FrCx) and whole blood (WB). These tissues differ in the extent of pathogenesis, including the level of splicing aberrations (Otero et al. 2021; Sznajder et al. 2020). Our analysis revealed that both diseased tissues show a global increase in circRNAs compared to non-DM control samples. However, their levels were not correlated with changes in parental gene expression or with the extent of aberrant alternative splicing (AAS). We found that mis-spliced CEs in DM1 and DM2 were not among the circularized exons. The unique genomic features of circRNA-producing loci that facilitate back-splicing did not include an enrichment of MBNL1 binding sites in introns flanking circularized exons, supporting earlier findings in DM1 skeletal muscles (Czubak et al. 2019a, b). Importantly, the pattern of AAS seen in linear RNAs is reflected in various circRNA isoforms. The large quantity of circRNAs also results from the use of cryptic exonic and intronic splice sites in back-splice junctions (BSJs), with intron-containing circRNAs being more frequent in WB. Overall, this study reveals that circRNAs, once considered splicing errors or by-products of canonical splicing, are dysregulated in various tissues from DM1 and DM2. However, their levels do not correlate with the splicing aberrations observed in linear RNAs, suggesting a potential independent regulatory mechanism underlying circRNA upregulation in myotonic dystrophy.

## Results

### Identification of CircRNAs in frontal cortex and whole blood from DM1 and DM2 patients

The global analysis of circRNAs in the FrCx and WB was conducted using previously published datasets of clinical samples from DM1 and DM2 patients, as well as non-DM controls (Otero et al. 2021; Sznajder et al. 2020) (Table S1). Details of the bioinformatics pipeline used for circRNA identification are provided under the Materials and Methods section. Briefly, the RNA samples submitted for sequencing were depleted of rRNA and HMR globin (in blood samples), and neither pre-selection for poly(A)-tailed species nor treatment with RNase R were performed, allowing for an unbiased quantification of both linear and circular RNAs. The transcriptome for each sample was profiled by paired-end, strand-specific capture RNA-seq, with sufficient depth to identify rare circular back-splicing events. To detect the circular BSJ reads from RNA-seq libraries, the CIRI2 pipeline was used (Gao et al. 2018). Custom R scripts were applied to filter the data based on three criteria: “Tier 1 (All)” (circRNAs confirmed by at least two BSJ reads in at least one sample, as defined by the default CIRI2 output), “Tier 2” (circRNAs confirmed by at least five BSJ reads in at least two samples), and “Tier 3” (circRNAs present in all or all but one sample of the analyzed dataset, regardless of the number of BSJs). The two-step filtering process of the CIRI2 output (All) aimed to identify high-confidence BSJs. Ultimately, circRNAs identified in the most stringent filtering category (Tier 3), were considered *bona fide* transcripts and included in the output files used for further analysis (Tables S2-S5). As shown in Figure S1, regardless of the filtering criteria, the greater number and diversity of circRNAs and their host genes were detected in the DM1 and DM2 samples from the WB, when compared to the FrCx. These genes produced either single or multiple circular transcripts, including their AS isoforms, with multi-circRNA genes (MCGs) being more abundant in the blood than in the cortex. This initial quantitative analysis revealed no positive correlation between the amount of circRNAs and the extent of AAS of linear transcripts, as described in earlier studies (Otero et al. 2021; Sznajder et al. 2020). According to these published data, DM1 and DM2-associated AAS (including CEs, mutually exclusive exons, alternative 5’ and 3’ splice sites, and intron retention) was predominantly detected in the brain of patients. In contrast, the blood from DM2 patients showed less pronounced splicing defects, and no splicing abnormalities were observed in the blood of DM1 patients.

**Fig. 1:**
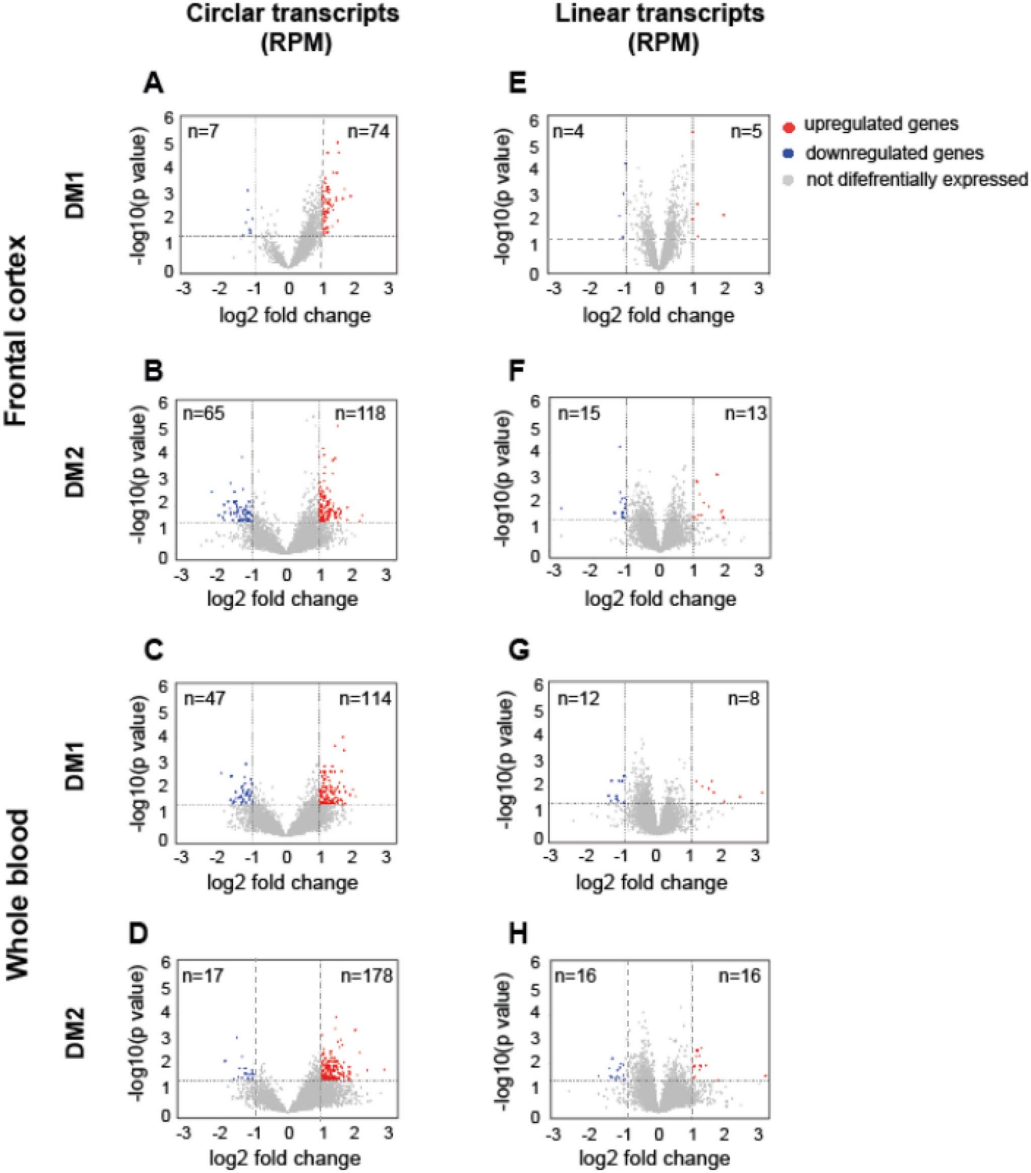
*The Prevalence of CircRNAs in DM1 and in DM2*. Volcano plots depicting differences in the levels of “high-confidence” circRNAs (Tier 3) and their linear host transcripts in DM1 and DM2 samples from FrCx (**A**, **B**, **E**, **F**) and WB (**C**, **D**, **G**, **H**). Each red dot (upregulated) and blue dot (downregulated) represent individual transcripts fulfilling the following criteria of expression change: p value < 0.05 and log2 fold change ≤ -1 or ≥ 1 (the thresholds indicated by dotted lines)

Differential abundance analysis of circRNA expression patterns between diseased and healthy controls was performed in two pairs: DM1 vs. control (sub-set 1) and DM2 vs. control (sub-set 2). The levels of individual circRNAs and their linear cognate transcripts were calculated as the number of BSJ reads and non-junction reads per million mappable reads (RPMs), respectively. This analysis revealed that diseased samples from both tissues exhibited a unique signature of circRNA expression levels, with specific subsets of transcripts being altered in DM1 and DM2 compared to controls. In particular, there was a global upregulation of circRNAs in both neuronal and blood tissues in both types of myotonic dystrophy. These results are detailed in Tables S2-S5 and graphically summarized in Fig. 1. As shown in Fig. 1, among the circular transcripts that exhibited differential expression in DM1 and DM2 (meeting the criteria of log2FC ≤ − 1 or ≥ 1, *p* < 0.05), 91.4% of circRNAs in the FrCx were upregulated in DM1 samples, and 64.5% in DM2 (chi-squared test, *p* < 0.0001) (Fig. 1A-B). Similarly, in WB, the upregulated circRNAs remains in the majority and consisted of 70.8% (in DM1) and 91.3% (in DM2) (chi^2^-squared test, *p* < 0.0001) (Fig. 1C-D). In all subsets, circRNA abundance was not correlated with the baseline expression of the parental genes (Pearson’s correlation coefficient R² < 0.1) (Fig. 1E-H). While no published data exist on circRNA analysis in DM2, our findings in DM1 are consistent with previously published reports from studies on skeletal muscle samples from DM1 patients and a mouse model of the disease (Czubak et al. 2019a, b; Voellenkle et al. 2019). Both studies highlight the lack of correlation between the levels of circRNAs and their corresponding linear transcripts. This suggests that in different human tissues with a DM1 genetic background, the variability in circRNA abundance cannot be directly explained by the expression levels of their parental genes. Instead, it may be influenced by intrinsic *cis*-regulatory elements and *trans*-acting factors, including regulatory splicing mechanisms and varying rates of circRNA stability and turnover in diseased versus control samples. Nevertheless, our study provides further evidence of circRNA dysregulation across various tissues in DM1 and DM2 patients. To validate our RNA-seq findings, we randomly selected a subset of circRNAs for validation in human brain and blood samples. We designed PCR assays with divergent primers for the circRNAs and convergent primers for their linear mRNA counterparts (Figure S2A; Table S6). The size of the circRNA-specific and corresponding linear amplicons was analyzed using agarose gel electrophoresis, and the predicted BSJs were confirmed through Sanger sequencing (Figure S2B-D). All 35 selected circRNAs were successfully validated in these assays.

### Characteristics of differentially expressed CircRNAs

The vast majority of identified circRNAs originated from annotated genes, which generated either single- or multi-circular species as documented in circATLAS (Tables S2-S5). In the FrCx, approximately 8% (in DM1) and 15% (in DM2) of all human protein-coding genes, estimated at 20,000 (Nurk et al. 2022), yielded both circular and corresponding linear transcripts. In contrast, in the WB, these genes comprised about 18% in both DMs. The circRNA genes were found on all chromosomes without any specific preference for their location (Figure S3). The Gene Ontology (GO) analysis of circRNA parental genes (https://david.ncifcrf.gov/home.jsp) revealed distinct functional enrichments. In the FrCx, genes corresponding to circRNAs upregulated in DM1 were enriched in terms related to protein transport, while in DM2, they were enriched in intracellular signal transduction and nervous system development (Figure S4A). In the WB, genes corresponding to DM1-upregulated circRNAs were enriched in transcription and metal ion binding, whereas in DM2, they were enriched in cell cycle and protein binding (*p* < 0.01) (Figure S4B). Notably, genes corresponding to downregulated circRNAs were not included in the functional association analysis due to their low number.

Based on the CIRI2 output, circRNAs were classified into four major categories: exonic, intronic, intergenic, and mixed (combinations of exonic + intronic, exonic + intergenic, and intronic + intergenic). Both the sense and antisense strands were equally utilized in circRNA generation (Figure S5). Among the differentially expressed circRNAs, the predominant category was exonic circRNAs, which accounted for approximately 90% of Tier 3 circRNAs in the FrCx and 80% in WB. These circRNAs contained either single or multiple exons from a given gene. The second most abundant category was exonic + intronic, which was more enriched in the WB (Figure S5 C-D). Notably, circRNAs of intergenic and mixed intergenic compositions were excluded from further analysis (they comprised less than 1% of the CIRI2 output). Based on circRNA coordinates, their lengths ranged from approximately 60 nucleotides (nt) to over 10,000 nt (Figure S6). The overall distribution of circRNA lengths was similar in both tissues between DM1 and DM2. The most represented length class was 200-1,000 nt (70–76%), with the majority being of exonic composition (99.5% in FC and 68% in WB) (Figure S6 B-E). Considering that the average length of an exon is approximately 200 nt, this suggests that a significant portion of circRNAs represents multi-exonic molecules, while single-exon circRNAs were found to be in the minority (3–6% of Tier 3 circRNAs in the FrCx and WB, respectively). The issue of circularized exon lengths remains a topic of ongoing debate. Some earlier studies have suggested that shorter exons are less likely to circularize, while others argue that smaller exons tend to form circles more readily (Zhang et al. 2014; Szabo et al. 2015). However, our data indicate that as circRNA length increases, the proportion of exonic circRNAs gradually decreases, with circRNAs of mixed composition becoming more prevalent in both DM1 and DM2 across both tissues. This trend was most pronounced in the WB, where mixed (exonic + intronic) circRNAs were most frequently found among transcripts longer than 1,000 nt (Figure S6 D-E). It is important to note that, due to the use of short-read RNA-seq data, our analysis focused on identifying multiple variants of circRNAs based on BSJ coordinates rather than their full lengths. As a result, this approach limits our ability to fully characterize the biogenesis and functional roles of longer circRNAs, particularly those that may involve complex combinations of exons and/or introns due to the AS.

### Features of CircRNA-producing loci

**Fig. 2:**
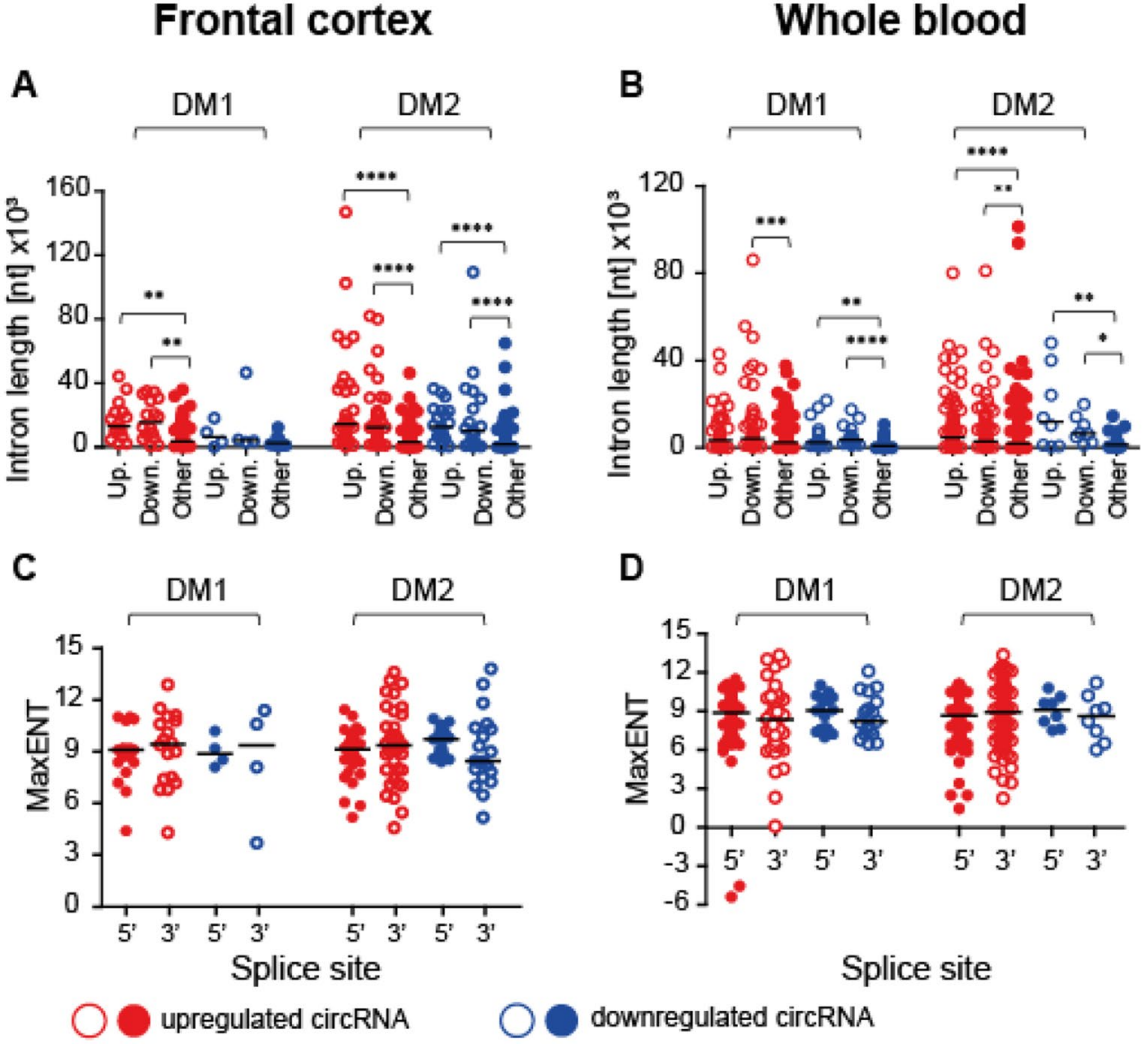
*Features of CircRNA-Producing Loci*. (**A**-**B**) The length of introns flanking BSJs in differentially expressed circRNAs (log2FC (≤-1.2 & ≥ 1.2) along with size of other introns in the FrCx (**A**) and WB (**B**). Red and blue opened and filled circles indicate, respectively, the features of introns in upregulated and downregulated circRNA genes; “up.” and “down.” indicate introns upstream and downstream of circular BSJs, whereas “other” depicts the other introns of circRNA genes away from BSJs; *p < 0.05, **p < 0.01, ***p < 0.001, ****p < 0.0001 (two-tail student’s *t*-test). (**C**-**D**) The maximum entropy (MaxEnt) score measuring the strength of splice sites at the 5’ and the 3’ ends of BSJs in individual circRNAs from the FrCx (**C**) and WB (**D**). Red and blue circles indicate, respectively, the MaxEnt in individual upregulated and downregulated circRNAs measured at the 5’ ends (filled circles) and 3’ ends (opened circles) of splice sites

Previous studies have suggested that circRNA formation is dependent on both *cis*-regulatory elements and *trans*-acting factors, particularly highlighting the sequence requirements in introns flanking BSJs. A critical role in this process has been attributed to intronic Alu repeats derived from short interspersed nuclear elements (SINEs) oriented in opposite directions. Disruption of these repeats has been shown to prevent circRNA formation (Jeck et al. 2013; Liang D and Wilusz JE, 2014; Ivanov et al. 2015). In light of this, we selected the top differentially expressed circRNAs and analyzed the intrinsic genomic features surrounding their BSJs. Using Repeat Masker (https://repeatmasker.org/), we screened for intronic SINEs. Our analysis of DM1 and DM2 samples from both the FrCx and WB revealed that the introns flanking circularized exons were significantly longer than those of non-circularized exons (Fig. 2A-B). These introns contained various Alu elements and exhibited more reverse complementary matches (RCMs) compared to introns from non-circRNA exons (Figure S7; Table S7). Interestingly, these intron features, including the structural stability of the RCMs (measured by the negative free energy value (∆G) for the complementary regions) (data not shown), were shared by both upregulated and downregulated circRNAs. This suggests that while RNA circularization requires similar *cis*-elements, the regulation of circRNA processing, including their stability, is further modulated by factors that differ between diseased and healthy conditions.

It has been suggested that AS is linked to exons that lack canonical splicing signals, and these “weak exons” have been considered a source of genetic susceptibility to splicing errors (de la Mata et al. 2003). To investigate whether this feature might influence splice site (“ss”) selection, leading to exon circularization, we analyzed the strength of the 5’ and 3’ splice sites of BSJs in individual circRNAs, along with the composition of dinucleotides at these junctions. We compared these results with features of other splice sites not involved in circRNA formation. The strength of “ss” was measured using the maximum entropy (MaxEnt) score, developed by Yeo and Burge (Yeo G and Burge CB, 2004). As shown in Fig. 2C-D, the average MaxEnt scores for differentially expressed circRNAs in DM1 and DM2 from the FrCx and WB ranged from 9.1 to 9.8, with no statistically significant differences between upregulated and downregulated circRNAs (*t*-test). Notably, the MaxEnt scores of the BSJs were not the weakest of the given gene when compared with its other “ss” (Figure S8 A). Additionally, for a few housekeeping genes that do not form circRNAs, the average MaxEnt scores were significantly lower (below 8.0) compared with those of circRNA genes (Figure S8 B). Overall, these results indicate that in DM1 and DM2 from both FrCx and WB, the strength of splice sites at BSJs is unlikely to be a driving factor in circRNA generation.

### Cryptic splice sites in exons and introns are involved in CircRNA formation in DM1 and DM2

The biogenesis of the vast majority of human exonic circRNAs occurs at the major U2 spliceosome at annotated “ss” flanked by canonical dinucleotides “GU” and “AG” at the 5′ and 3′ ends of an intron, respectively (Zhang et al. 2011, 2022). However, circRNAs processed by the minor U12 spliceosome have also been reported, with BSJs found at unannotated “ss” (Szabo et al. 2015). Our analysis of individual circRNAs from DM1 and DM2 revealed that the majority of BSJs were at canonical donor/acceptor positions, whereas approximately 10–15% (respectively, in the FrCx and in the WB) were located at unannotated “ss” with either canonical or non-canonical dinucleotides (Tables S8 and S9). These BSJs were detected both in exons (ie-BSJs) and in introns (ii-BSJs). The ie-BSJs were often characteristic of the last exons of circRNA genes. Based on the Alternative Splice Site Predictor (ASSP) tool (http://wangcomputing.com/assp/) (Wang M and Marin A, 2006), they were categorized as cryptic, constitutive, or unclassified donors or acceptors. Thus, splice sites in exons are involved in back-splicing, leading to the production of circRNAs that contain partial exon sequences. Interestingly, we identified that cryptic “ss” contributing to circRNA biogenesis are located either in the last coding exons (e.g., hsa_CPNE1_0001) or in the 3′-UTR (e.g., hsa_ZNF250_0004) (Figure S9; Table S8). Based on previously published human transcriptomics data, the non-canonical splicing signals inside annotated protein-coding exons were attributed to cryptic introns called exitrons (Marquez et al. 2015). Exitrons are unique in that they have both protein-coding (exon) and splicing (intron) potentials while also possessing canonical splice-site signals. However, the first exons of circRNA genes have also been identified with non-canonical “ss,” and these were classified as cryptic acceptors (Table S8). Thus, terminal exons, which lack their 3′ or 5′ splicing signals and would normally prevent them from circularizing, overcome this obstacle and become donors or acceptors of new cycles of the AS.

The second type of unannotated “ss” was found in introns and was typical either of purely intronic (e.g., hsa-ROBO2_0044) or exonic-intronic circRNAs (e.g., hsa-RASGRF2_0029) (Table S9). The latter group was the majority, regardless of the subsets analyzed, and this category of circRNAs predominates in the blood compared with the cortex. Over 61% of intronic circRNAs have their “ss” in the first or second introns, whose lengths range from a few thousand to over 1 million nucleotides. About 30% of these circRNAs have cryptic “ss” marked by non-canonical dinucleotides, with MaxEnt scores ranging from 2.45 to 14.43 (Table S9). Further characteristics of circRNAs with canonical and non-canonical “ss” involved in the formation of BSJs revealed significant differences in their levels, including those of their host linear transcripts. Overall, circRNAs originating from the utilization of unannotated splice sites were less abundant compared to those with canonical donor/acceptor “ss” (Figure S11). This observation suggests that the biogenesis of different types of circRNAs is influenced by divergent pathways and transcript isoforms, with ongoing experiments aimed at validating this hypothesis (Mroczko-Młotek A, manuscript in preparation).

Altogether, these results indicate that the utilization of cryptic splice sites in specific segments of circRNA genes contributes to their biogenesis in different tissues from DM1 and DM2. Cryptic “ss” in introns lead to the enrichment of the circRNA population containing partial intronic sequences, which are predominantly identified in blood. This suggests that circRNA formation involves tissue-specific splicing mechanisms that use both canonical and cryptic splice sites, warranting further investigation.

### Circularized exons are not among cassette exons aberrantly spliced in DM1 and DM2

**Fig. 3:**
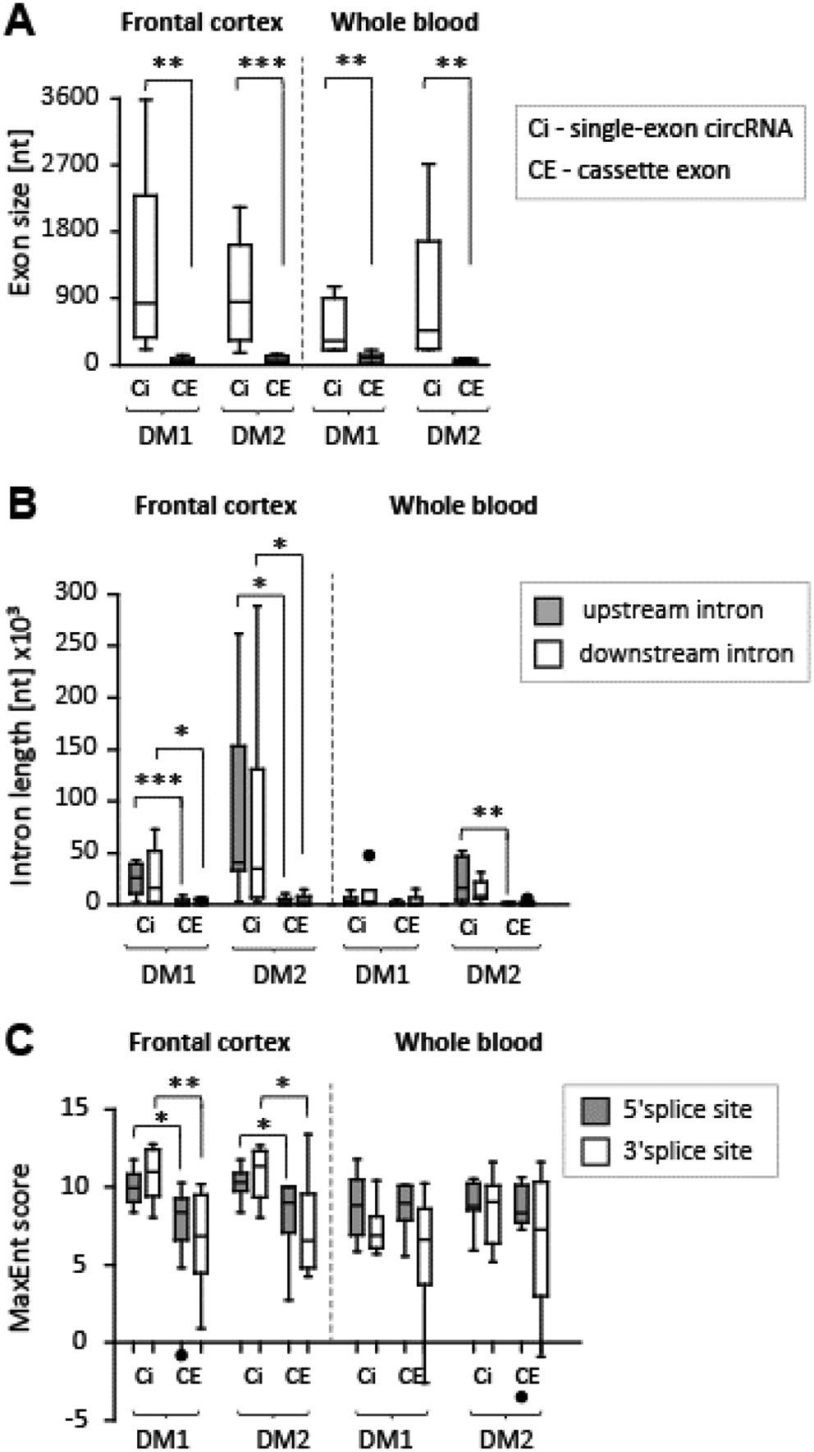
*Features of Single-Exon CircRNAs and Misspliced Cassette Exons*. The comparison of exon size (**A**), its flanking introns lengths (**B**), and splice sites strength (MaxEnt) (**C**) between single-exon circRNAs and CEs aberrantly spliced in the FrCx and WB (based on published results from Otero et al. 2021; and Sznajder LJ et al., 2020). *p < 0.05, **p < 0.005, ***p < 0.0005 (two-tail student’s *t*-test)

More than 95% of genes in the human genome are alternatively spliced, and one of the most common types of AS events is CE, also known as exon skipping (Dvinge H et al., 2015). In vitro and in vivo studies in flies and vertebrates have identified several characteristic features of CEs that differentiate them from constitutive exons. Specifically, CEs are shorter than constitutively spliced exons, have longer flanking introns, lower GC content, and weaker splice sites (Sorek et al. 2004; Rukov et al. 2007; Bell et al. 1998; Fox-Walsh et al. 2005; Kim et al. 2007; Gelfman et al. 2012). These associations suggest that genome architecture contributes to variations in AS types and frequencies. However, the enrichment of *trans*-acting factor binding in introns flanking the alternative exons also influences their splicing fate, as demonstrated in DM1 and DM2 studies (Savkur et al. 2001; Wang et al. 2019). Thus, we aimed to determine whether the above features also characterize exons that escape forward splicing by forming single-exon circRNAs (SE-circRNAs). SE-circRNAs comprised about 3–5% of the highly confident circRNAs identified in the FrCx and WB. We compared this result with the aberrant splicing of CEs described previously in these two tissues (Otero et al. 2021; Sznajder et al. 2020). First, we found no common exons between SE-circRNAs and CEs that were aberrantly spliced in DM1 and DM2 for a given gene. Second, our results showed a consistent relationship between gene architecture and the occurrence of either SE-circRNAs or CEs; however, these events did not share similar characteristics (Fig. 3). In particular: (i) exons of SE-circRNAs were always significantly longer than CEs; (ii) SE-circRNAs were flanked by much longer introns, mostly in the FrCx; and (iii) the strength of the 5′ and 3′ “ss,” as measured by MaxEnt scores, was significantly higher in SE-circRNAs from the FrCx, whereas in WB, there was no such diversity. Thus, in the FrCx from DM1 and DM2, heterogeneity in the splice sites influenced the selection of an AS type, with exons having more poorly defined intron-exon boundaries being more susceptible to CE events, whereas those with stronger “ss” were more likely to undergo circularization.

In searching for factors associated with exon circularization, we also analyzed the binding signature of splicing factors in regions flanking BSJs of SE-circRNAs and compared them with those of CEs aberrantly spliced in DM1 and DM2 (Otero et al. 2021; Sznajder et al. 2020). Based on published data obtained from CLIP analysis of MBNL1 and CUGBP1 proteins, their binding to upstream or downstream regions of alternative exons stimulates antagonistic effects (Wang et al. 2012). While MBNL1 promotes alternative exon exclusion when binding to the cassette and upstream of it, CUGBP1 achieves the same effect when binding downstream (Masuda et al. 2012). This scenario is disrupted in myotonic dystrophies due to diminished functional levels of MBNL1 and upregulation of hyperphosphorylated CUGBP1, which is predominantly found in DM1 (Kuyumcu-Martinez et al. 2007; Wang GS and Cooper TA, 2007). Using the SpliceAid database of human splicing factors and their RNA-binding sites (Giulietti et al. 2013), we searched for binding enrichment of these proteins in three distinct regions: the circularized exon itself, and 250 nucleotides downstream and upstream in the flanking introns. For MBNL1, we identified 5-mer motifs (YGCY), including UGCUU and GCUGC, while for CUGBP1, the motif was UG-rich (Wang et al. 2012; Lambert et al. 2014). Among the differentially expressed SE-circRNAs from the FrCx and WB, we did not find exons with a strong signature for MBNL1 binding characteristic of exon exclusion. However, in the cortex from DM1, we identified enrichment of CUGBP1 binding in the introns flanking BSJs of some upregulated SE-circRNAs (e.g., hsa-RC3H1_0001 and hsa-DCLK2_0001 had 96 and 62 CUGBP1 binding sites, respectively, within 250 bp downstream of the circularized exons). Interestingly, elevated levels of hsa-RC3H1_0001 were also detected in DM1 skeletal muscle, suggesting that upregulation of CUGBP1 in these two DM1 tissues may affect the biogenesis of some circRNAs. In support of this assumption, no statistically significant differences in the levels of these two circRNAs were found in the WB or in any tissues from DM2. The potential involvement of CUGBP1 in RNA circularization is currently under investigation (Srinivasan A, manuscript in preparation).

Altogether, these results reveal distinct features of circularized exons that differentiate them from constitutive and alternatively spliced exons in DM1 and DM2. The involvement of MBNL1 in the biogenesis of circRNAs, which was questioned in a previous study of DM1 skeletal muscles, is supported by the current analysis. However, our preliminary results suggest that the circularization of some RNAs could be influenced by CUGBP1 protein, which is primarily implicated in DM1 pathogenesis.

## Variety of CircRNA species

### Single-circRNA genes and Multi-circRNA genes

**Fig. 4:**
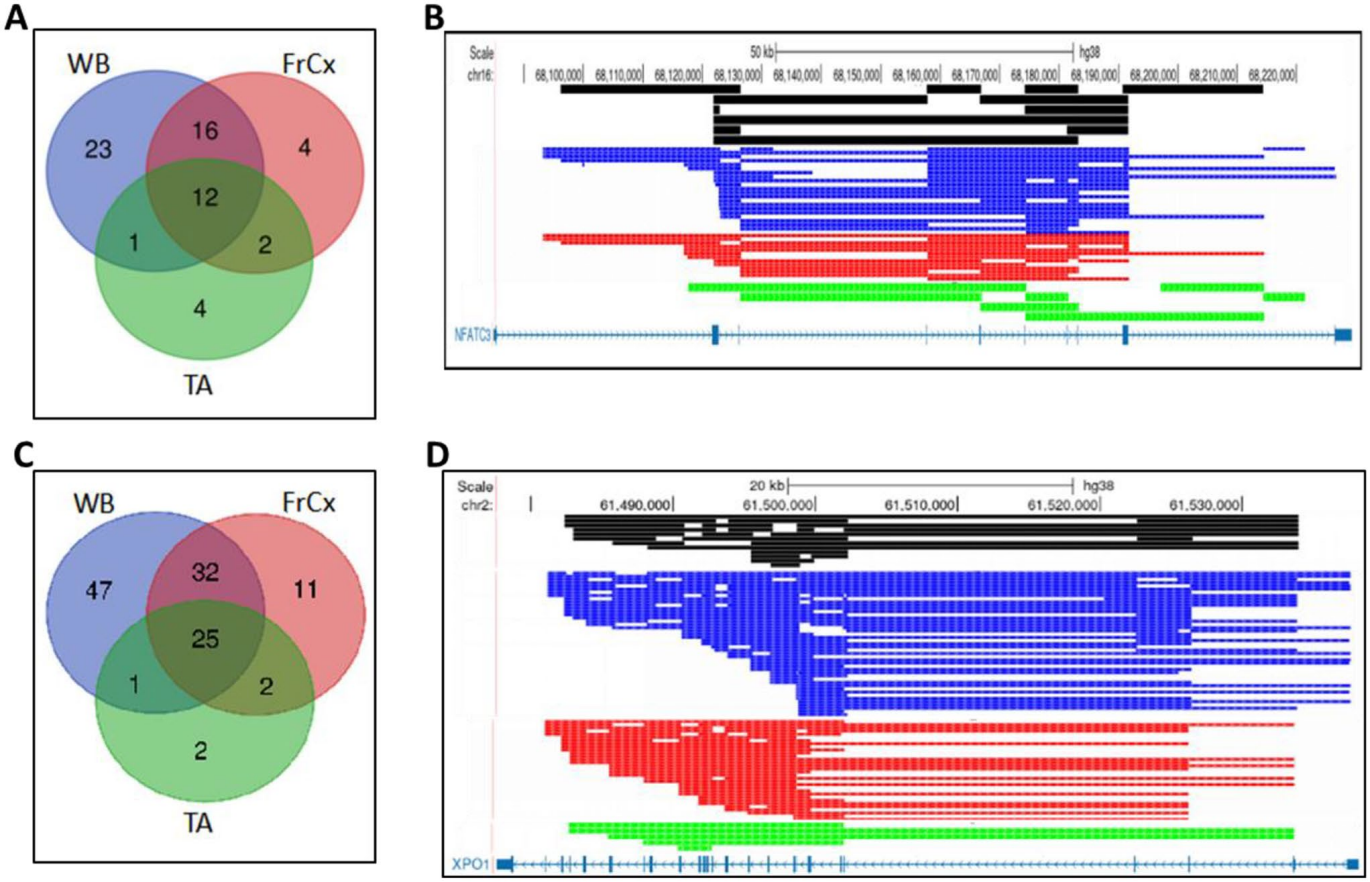
*CircRNA Isoforms of the Multi-CircRNA Genes NFATC3 and XPO1 in Different Tissues from DM1 Patients*. Venn diagram depicting the tissue-specific and common circRNAs of NFATC3 (**A**) and XPO1 (**C**) identified in whole blood (WB), frontal cortex (FrCx), and *tibialis anterior* (TA). (**B** and **D**) the distribution of circular transcripts common for all the tissues (black) and tissues-specific, i.e., blue (in WB), red (in FrCx), and green (in TA)

Next, we found that individual genes generate either single or multiple circRNA species, including their isoforms, which arise from the AS of linear pre-mRNA. These genes were grouped into single-circRNA genes (SCGs) and multi-circRNA genes (MCGs), and they belong either to tissue-specific or ubiquitously expressed categories (https://fuma.ctglab.nl/). In the FrCx from DM1 and DM2, these two groups were represented nearly equally: 48% (SCGs) and 52% (MCGs), whereas in the WB, they consisted of 35% and 65%, respectively (Figure S12; Tables S2–S5). The top MCGs hosted more than 10 distinct circRNAs, and in the cortex, they comprised about 3% of all circRNA-generating genes, while in the blood, they represented ~ 23%. One extreme example was SOX6 (SRY-box transcription factor 6; ENSG00000110693) from DM2 blood, which generates more than 400 different circular species, whereas only 18 linear transcripts (9 protein-coding and 9 non-coding processed transcripts) were recorded for this gene in the Ensembl database (Table S5). In contrast, in the FrCx, the highest number of circular forms was generated from HERC1 (probable E3 ubiquitin-protein ligase; ENSG00000103657), which could express over 100 circRNAs, while only 15 various linear RNAs (6 protein-coding and 9 non-coding transcripts) were observed (Figures S13; Table S2). Overall, for the top MCGs, circular transcripts were more prevalent than their linear isoforms. Interestingly, these circRNAs were neither disease- nor tissue-specific. In addition to the FrCx and WB, we also found them in skeletal muscle (*tibialis anterior*). It is not unusual that about 50% of circRNAs from different tissues share genomic locations of their BSJs for a given MCG (Fig. 4). This suggests the existence of intrinsic hotspots of circRNA biogenesis that are recognized and utilized by transcription/splicing machineries across various tissues (Memczak et al. 2015).

Searching for factors affecting the observed vast quantity of circRNAs, we analyzed genes’ total lengths and their expression levels for groups of SCGs and MCGs. Transcriptomic expression data for DM1 and DM2 were retrieved from a previous publication (Otero et al. 2021). As shown in Figure S14 A-D, no correlation was found between these two factors and the number of circular species (Pearson coefficient R² < 0.06), supporting the notion that the number of splice sites in genes and the variety of their transcripts do not determine the magnitude of circRNA production. Interestingly, for some MCGs, there was a negative correlation between their expression levels and the quantity of circRNAs. This feature was particularly evident in blood, where exceptionally high numbers of circRNAs per gene were observed compared to the cortex. The most striking example was KAT2B, which could transcribe into > 200 circRNAs in the WB but only 24 in the FrCx, despite its marginal expression in blood. A similar trend was found for STRN3 (131 and 16 circRNAs, respectively, in WB and FrCx) and for CAMKMT (193 and 10 circRNAs, respectively, in WB and FrCx) (Figure S15). Notably, in the WB, over 70% of these circRNAs are of intronic and exonic-intronic structures, originating from the utilization of cryptic splice sites (Tables S2–S5).

### CircRNA isoforms

**Fig. 5:**
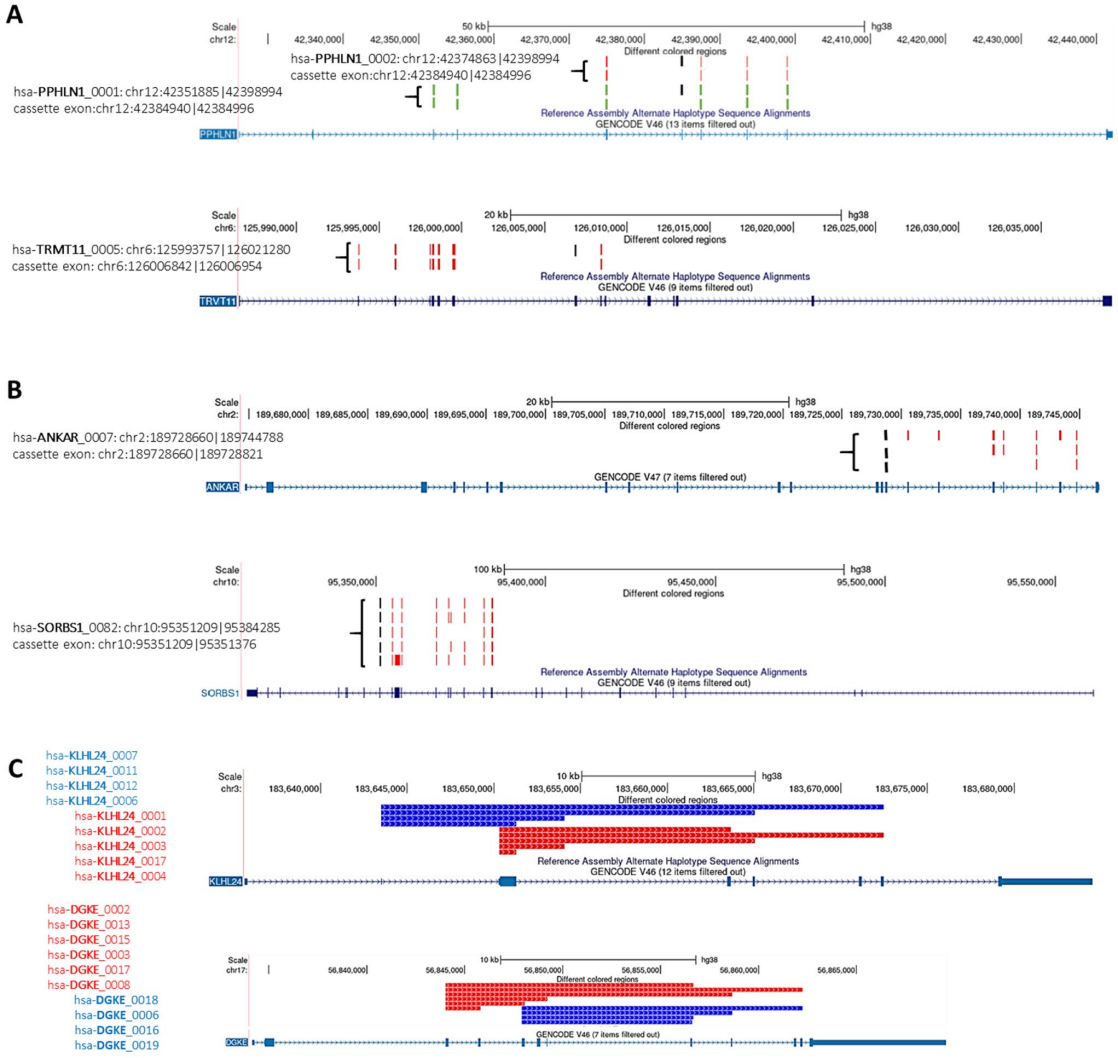

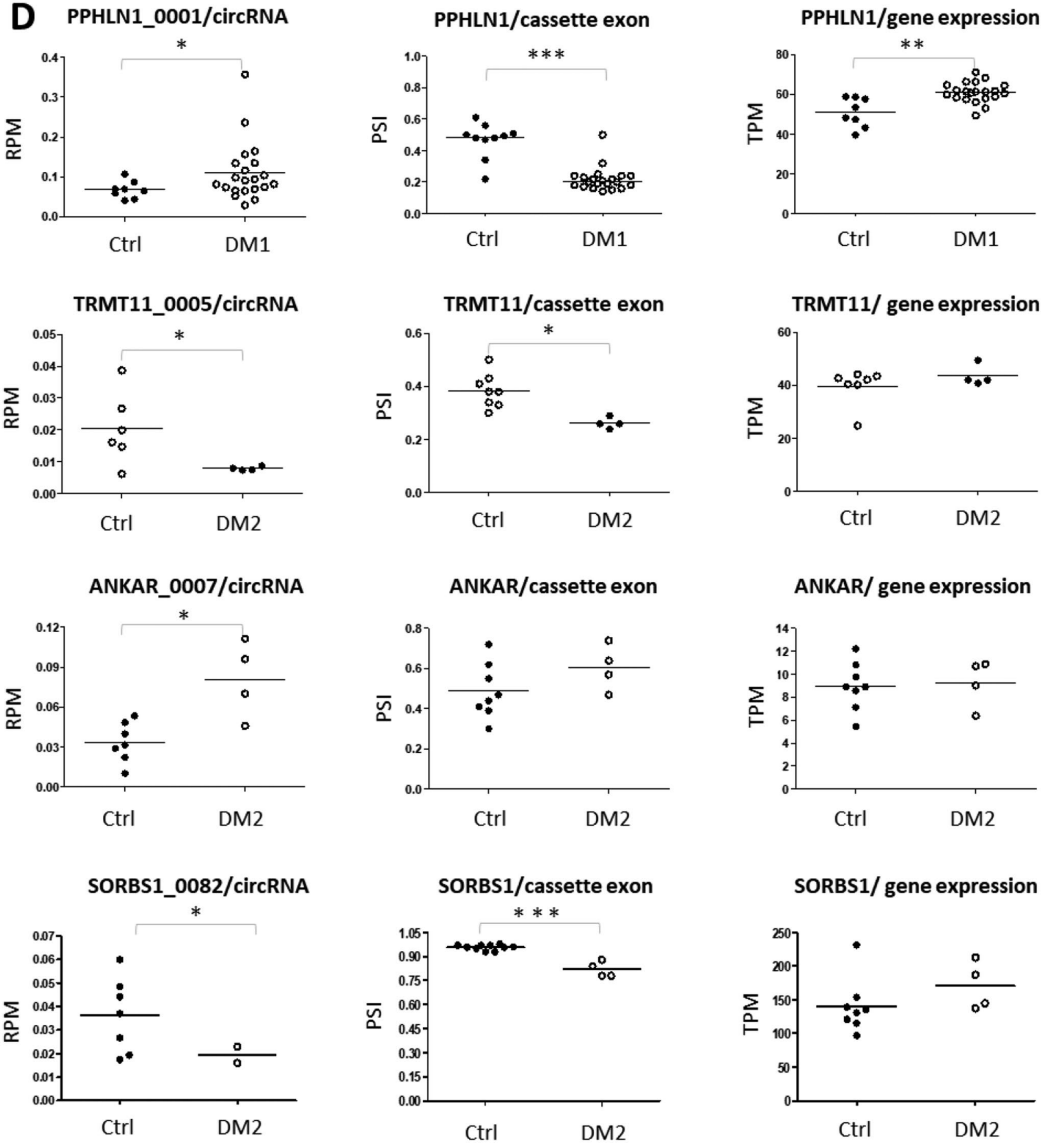
*Alternative Splicing as a Source of CircRNA Isoforms*. (**A**) Schematic illustrations of circRNA isoforms of PPHLN1 and TRMT11 due to mis-splicing of CEs determined previously in the FrCx by Otero et al. (2021) and shown in (**D**). (**B**) CircRNA isoforms of ANKAR and SORBS1 due to the differential content of alternative exons. CircRNAs in (**A**) and (**B**) have consistent BSJs but different compositions. Black bars, CEs; red and green bars, other exons of circRNA genes. (**C**) Representative examples of circRNA isoforms from KLHL24 and DGKE due to different utilization of 5’ donor splice sites and consistent acceptor splice sites in BSJs. (**D**) The plotted results of circRNA levels (shown in **A** and **B**) along with linear transcript levels and percent spliced in (PSI) of the CEs (black bars in panels **A** and **B**). *p < 0.05; **p < 0.001; ***p < 0.0001

The vast diversity of circRNA species originating from different regions of the MCGs does not fully reflect their entire population as described by the circATLAS IDs. In fact, various circRNA isoforms, which carry the blueprints of the AS from linear transcripts (including CEs), are found in RNA-seq data from CIRI2 output in DM1 and DM2 (Li et al. 2022; Dong et al. 2019). These isoforms share identical BSJ locations but differ in their internal composition of exons and/or introns (Fig. 5A, B). Among these circRNA isoforms, we identified two groups: (i) isoforms generated due to mis-splicing of CEs previously identified in transcriptomic studies of FrCx and WB from DM1 and DM2 patients (Otero et al. 2021; Sznajder et al. 2020) (Fig. 5A, D) and (ii) isoforms with consistent presence of the CEs but differential content due to other alternative exons of circRNA genes (Fig. 5B, D). About 39% of all circRNAs identified in the FrCx (from both DM1 and DM2) were found to encompass one of the mis-spliced CEs detected previously by Otero and colleagues (Otero et al. 2021); whereas ~ 33% of circRNAs identified in the neuronal tissue from both disease conditions belong to the second category of isoforms (Figure S16 A). In addition, circRNA isoforms may represent events originating from the utilization of alternative back-splice site selection, resulting in species with different 5′ splice donors or 3′ splice acceptors (Fig. 5C). These isoforms comprised nearly 39% and 22% of circRNAs identified from MCGs in DM1 and DM2, respectively (Figure S16 B). Their presence suggests the existence of hotspots in circRNA genes that are more prone to back-splicing than other loci.

Altogether, these results show a lack of correlation between the variety of circRNAs and gene size, as well as their expression levels. While most circRNAs arise from multi-exonic genes and exhibit diversity through AS, including CEs, the full extent of AS in circRNAs relative to linear mRNAs has not yet been fully explored.

## Discussion

Developments in transcriptome sequencing and analysis have revealed a remarkable prevalence of unconventional or non-canonical splicing mechanisms, ranging from the recognition of atypical splice sites to changes in the usual order of splicing (Wang et al. 2008; Braunschweig et al. 2014; Barbosa-Morais et al. 2012). Research over the last decade has shown that, in some cases, pre-mRNA splicing does not follow its canonical order and occurs in a reversed orientation, called back-splicing, to produce a circRNA (Zhang et al. 2014; Memczak et al. 2013). Thousands of circRNAs derived from protein-coding genes have been identified in various tissues across different species. Although these molecules are widespread and suggested to play essential biological roles, the exact mechanism behind their formation remains unresolved. Various studies have indicated that circRNA levels often rise in certain diseases, suggesting a link to their pathomechanism. In our research, we observed a global upregulation of circRNAs in individuals with DM1 and DM2, both in the FrCx and WB, corroborating findings from previous studies conducted on DM1-specific skeletal muscle tissues (Czubak et al. 2019a, b; Voellenkle et al. 2019). Thus, RNA circularization enriches the profile of the AS typical of DM1 and DM2. Interestingly, we did not observe significant changes in the overall levels of linear mRNAs originating from the loci that produce circRNAs, indicating that their upregulation is likely due to a specific regulatory mechanism affecting their formation, rather than changes in gene activity or mRNA production. For many genes, their negligible expression levels were negatively correlated with a plethora of circRNA species, which challenges the common notion that these RNAs result from splicing errors.

Numerous *cis*-elements and *trans*-acting factors have been proposed to play a role in the AS outcomes, including the generation of covalently closed circRNA molecules (Liang D and Wilusz JE, 2014; Zhang et al. 2014; Conn et al. 2015). One such factor involves the genetic features of flanking introns, as well as the composition and strength of acceptor and donor splice sites (Jeck et al. 2013; Liang D and Wilusz JE, 2014; Wilusz 2015). Motif analysis of the exon-intron boundaries revealed that over 99% of circRNAs identified in our study were flanked by the canonical splicing motif gu/ag, typically recognized by the major spliceosome. This is in agreement with previous publications, as the majority of exonic circRNAs deposited in the circATLAS exhibit this motif (Szabo et al. 2015; Rybiczka-Tešulov et al. 2024; Zhou et al. 2024). Among cryptic acceptor/donor splice sites in introns and exons, the BSJs were also enriched for these consensus motifs. The AS was also modelled based on the strength of the 5′ and 3′ splice sites, with the non-canonical usage of “weaker” sites potentially facilitating circRNA generation. However, our analysis of the strength at BSJs did not conform to this rule, and there was no significant difference in the MaxEnt scores between circular and other splice sites. Overall, these results indicate that in FrCx and WB from DM1 and DM2, neither the upstream nor downstream dinucleotides flanking BSJs, nor the strength of the splice sites, are likely to be the driving factors of circRNA generation. Based on published reports, alternative mechanisms could also facilitate the splicing of exons adjacent to long introns, and this feature was considered when differentiating between constitutive and alternative exons. Elongated intronic sequences are also characteristic of circular BSJs, and their presence plays a role in the efficiency of back-splicing, mostly through inverted complementary sequences and the binding of splicing factors (Jeck et al. 2013; Cao 2021). Our analysis revealed that introns bordering circRNA-producing exons in the FrCx and WB were longer than other introns; they contained various Alu elements and many more RCMs. However, these features were common for all differentially expressed transcripts from DM1 and DM2, suggesting that the abundance of circRNAs in both diseased and healthy conditions is influenced more by post-transcriptional factors than by strictly *cis*-elements. Moreover, comparison of aberrant alternative splicing of CEs (Otero et al. 2021; Sznajder et al. 2020) with SE-circRNAs revealed no common exons, and the aberrantly excluded CEs were not subjected to circularization. Importantly, this scrutiny led to the identification of a new category of exons, whose genomic features differed from those of constitutive and alternative exons. Additionally, as shown by in silico analysis, this new category of exons did not exhibit enriched binding of splicing factors, such as MBNL proteins, in their flanking introns, as previously reported for some exons aberrantly skipped in DM1 and DM2. This may suggest that the regulation of circularized exons could involve distinct mechanism(s), underscoring a novel regulatory complexity in disease-related splicing events.

According to the current release of Ensembl, the human genome is estimated to contain over 44,000 genes, just over 20,000 of which encode proteins (Piovesan et al. 2019; Howe et al. 2021). In our analysis, as many as 8–15% (in FrCx) and 18% (in WB) of the protein-coding genes were found to yield both linear and circular transcripts. Among these genes, there were SCGs and MCGs, with the latter often generating many more circRNAs than linear transcripts. The vast diversity of back-splicing products was enriched by the presence of isoforms derived from the blueprint of pre-mRNA alternative splicing of CEs. These isoforms represented events with consistent BSJs but different internal compositions, and in the FrCx, nearly 40% of all circRNAs from DM1 and DM2 were isoforms due to the presence or absence of CEs, as shown in earlier studies (Otero et al. 2021). Research in many laboratories has demonstrated that over half of transcribed loci can generate multiple circRNA isoforms through AS, with significant implications for cancer and other disorders. While most human circRNAs come from MCGs and exhibit diversity through AS, including CEs and/or retained introns, the full extent of AS in circRNAs relative to linear mRNAs has yet to be fully explored. The generation of different types of circRNAs in DM1 and DM2 from both analyzed tissues was also a result of the utilization of cryptic donor/acceptor splice sites within exons and introns, leading to the production of circRNAs that contain partial exon and/or intron sequences. Of particular interest are intronic circRNAs, as their mechanism of generation remains highly ambiguous. Previous reports suggested that these RNAs could originate from inefficient debranching of intronic lariats (Zhang et al. 2013). However, it is also plausible that they are generated by other, as-yet unknown mechanisms. One potential mechanism is non-canonical splicing of introns via recursive splicing, in which long introns are removed in a stepwise fashion through the use of intermediate intronic sites (Sibley et al. 2015; Kelly et al. 2015; Burnette et al. 2005). The presence of long introns in vertebrates, particularly in humans, raises important questions about how the spliceosome distinguishes between genuine and cryptic splice sites in vast intronic regions. These long introns, filled with numerous cryptic or pseudo-splice sites, may play a significant role in circRNA formation. Cryptic splice sites, often silenced by splicing silencer motifs, might occasionally escape repression or be activated under specific conditions, leading to the formation of circRNAs from intronic sequences. The diversity and abundance of cryptic splice sites suggest that intronic circRNAs could arise when these sites are mistakenly recognized by the spliceosome or when regulatory factors like SR and hnRNP proteins influence splice site selection. Further research is needed to uncover the specific mechanisms driving intronic circRNA formation, particularly how cryptic splice sites and regulatory proteins impact this process.

## Materials and methods

### Samples used for second-generation sequencing

Publicly available RNA-seq data were retrieved from the Gene Expression Omnibus (GEO) database and are listed in Table S1. The global analysis of circRNAs was performed in adult human FrCx and WB using, respectively, RNA-seq data sets generated by Otero et al. (2021) (GSE157428) and by Sznajder LJ et al. (2020) (GSE138691). The samples of FrCx were obtained from post-mortem frozen brains and included 21 DM1, 4 DM2, and 8 non-DM controls; they ranged in age from 39 to 83 and were split across both sexes (Table S1). The tissue samples were homogenized in TRIzol, followed by the Direct-Zol RNA Miniprep kit with DNase I treatment. RNA-seq libraries were constructed using the NEBNext Ultra II Directional RNA library prep kit for Illumina, with rRNA depletion followed by strand-specific RNA-seq preparation, and samples were sequenced using the Illumina NextSeq 500 v2 [33]. Whereas the WB included equal numbers of DM1, DM2, and non-DM samples, which represented both sexes and ranged in age from 23 to 60 (Table S1). Total RNA was extracted from the blood containing white and red blood cells, plasma and platelets. For the sample collection, total RNA from DNA/RNA Shield Blood Collection Tubes was isolated with TRIzol Reagent and the Direct-Zol RNA MiniPrep Kit with DNase treatment according to the manufacturer’s protocol (Zymo Research). The rRNA-depleted and globin-depleted strand-specific RNA-seq libraries were prepared using the KAPA Stranded RNA-seq Kit with RiboErase HMR Globin (Kapa Biosystems) per manufacturer’s instructions, and sequencing was performed using an Illumina NextSeq 500 (Sznajder LJ et al. 2020. The sequencing depth of RNA samples from, respectively, FrCx and WB exhibited, on average, 130 million and 120 million paired-end reads of 75 bp per sample, thus being sufficient for the identification of not only gene expression but also rare back-splicing events. Commercial sequencing providers suggest > 40 million reads per sample as sufficient for circRNA detection. Of note, the circRNAs originating from the *tibialis anterior* (TA) were previously analyzed by Czubak K et al. (2019). The RNA sequencing data used for circRNA identification were generated by Wang et al. (2019) (GSE86356) and include 21 DM1 and 6 non-DM biopsy samples, ranging in age from 15 to 69 and representing both sexes.

### Bioinformatics analysis of RNA-sequencing data

The raw sequencing files were downloaded in FastQ format (Cock et al. 2010). After trimming the Illumina adapters, filtered reads were aligned to the human reference genome GRCh38 using the BWA-MEM aligner with a specified alignment score threshold T = 19 (version 0.7.12, Wellcome Trust Sanger Institute, Wellcome Trust Genome Campus, Cambridge, UK). The reads mapped to either 5’ or 3’ splice sites in a reverse order were qualified as BSJs. CIRI2 was used to identify these junctions, which are characteristics of circRNAs (Gao et al. 2018). By default, the algorithm yields all circRNAs regardless of their junction read counts, and such “no stringency” output was named “Tier 1” (all). Next, the all identified circRNAs were filtered using two different filtration options, giving rise to a “Tier 2” (indicating circRNAs present in at least two samples of the compared group with the sum of reads in all samples of at least 5), and a “Tier 3” (indicating circRNAs present in all or in all but one sample in the analyzed set regardless of their junction read number). Then, circRNAs identified in the Tiers were collected, normalized to each library size, and quantified using a custom R script, and their expression levels were calculated as a number of circRNA-specific reads per million mappable reads (RPM). The output of Tier 3 was used to assess the frequency of circRNAs that were differentially expressed using a t-test in a custom R script. All the analyses were performed by comparing diseases samples with controls. Summary statistics for each set are collected in Tables S2-S5.

### Analysis of features of circRNA-producing loci

Sequences of exons and introns of circRNA genes were extracted from the GRCh38 UCSC Genome Browser. The following features were characterized: sequence and architectural parameters at the gene level and alternative splicing event level. At the gene level, we analyzed the total number of exons and introns of circRNA genes, their lengths, and the positions of BSJs within the gene sequence. Control (other) introns were randomly selected from the remaining introns of circRNA host genes. The lengths of upstream and downstream flanking introns of circRNA genes and other introns were calculated separately. At the alternative splicing event level, we recorded total expression levels of circRNA genes and aberrant alternative splicing in DM1 and DM2 samples from FC and WB determined in the previous studies (Otero et al. 2021; Sznajder et al. 2020).

The number and distribution of SINEs (short interspersed elements) in upstream and downstream introns flanking circRNA BSJs were determined using the UCSC Genome Browser’s Repeat Masker track. To identify the reverse complementary matches in a pair of flanking introns, the alignment of the sense- and antisense-oriented SINE sequences was performed using MegaBLAST (https://github.com/chenying2016/queries) (Chen et al. 2015). The RNAfold online tool (http://rna.tbi.univie.ac.at/cgi-bin/RNAWebSuite/RNAfold.cgi) was employed to predict RNA structures and to calculate the thermodynamic properties (ΔG values) of aligned RCM sequences.

To estimate the strength of the 5′ and 3′ “ss,” the maximum entropy (MaxEnt) score algorithm (http://genes.mit.edu/cgi-bin/Xmaxentscan_scoreseq.pl) was used (Yeo G and Burge CB 2004). This model assigns a log-odd ratio (MaxEnt score) for the 5′ “ss” (9 bp − 3 from the exon and 6 from the intron) and the 3′ “ss” (23 bp − 20 from the intron and 3 from the exon). The composition of dinucleotides at circular BSJs was determined using the UCSC Genome Browser and visualized with the WebLogo 3 tool (https://weblogo.berkeley.edu/logo.cgi).

### Analysis of binding motifs of MBNL1 and CUGBP1 in introns flanking BSJs

In silico analysis of the signatures of RNA-binding proteins was performed with the SpliceAid database of human splicing factors and their RNA-binding sites (Giulietti et al. 2013). Intronic sequences from 3 distinct regions of differentially expressed circRNAs were assessed: 250 bases upstream and downstream of the circularized exon(s), including the circularized exon(s). For MBNL1, we enumerated 5-mer motifs (YGCY), including UGCUU and GCUGC, whereas for CUGBP1, it was an UG-rich motif (Wang et al. 2012; Masuda et al. 2012). For the motif analysis of aberrantly spliced CEs in DM1 and DM2 from the FrCx and WB, we used splicing data from original publications by Otero et al. (2021) and Sznajder LJ et al. (2020).

### CircRNAs validation with RT-PCR and sanger sequencing

For circRNAs and linear host transcript validation, we used RNA samples obtained from human blood and brain (Telethon Foundation) and human DM1 and DM2 B-lymphocytes (Coriell Institute). The cells were cultured in DMEM supplemented with 10% FBS and 1x antibiotic-antimycotic (Thermo Fisher), and total RNA was isolated from the cell pellet with TRIzol reagent (Life Technologies) per the manufacturer’s instructions. For all samples used, RNA concentration was determined using a spectrophotometer (Thermo Scientific). cDNA was generated using Superscript III reverse transcriptase (RT) (Invitrogen) following the manufacturer’s instructions. 0.5 µg RNA was used as a template per 20 µl RT reaction. For circRNAs, amplification primers followed a divergent design in which the 5′ primer was placed at the 3′ end of the exon of interest and the 3′ primer was placed at the 5′ end. These primers were designed manually or using the CircInteractome web tool (https://circinteractome.nia.nih.gov/Divergent_Primers/divergent_primers.html). For linear RNA amplification, standard convergent primers were designed using the NCBI primer tool. Primer sequences are listed in Table S6. PCR was performed using Taq DNA polymerase (Promega) following the manufacturer’s instructions. For further circRNA validation, we performed Sanger sequencing.

### Statistical analysis

All statistical analyses were performed using Prism v. 5.0 (GraphPad, San Diego, CA, USA), Social Science Statistics (www.socscistatistics.com), or custom R scripts for the quantification and normalization of circRNAs. The R scripts were executed in RStudio 2023.09.1 + 494, and the following packages: tidyverse, dplyr, rtracklayer, GenomicRanges, and ggplot2 were used for analysis and plots. All p-values lower than 0.05 were considered statistically significant. All human genome positions indicated in this report refer to the GRCh38/hg38 human reference sequence.

## Electronic supplementary material

Below is the link to the electronic supplementary material.

Supplementary Material 1

Supplementary Material 2

Supplementary Material 3

Supplementary Material 4

Supplementary Material 5

Supplementary Material 6

Supplementary Material 7

Supplementary Material 8

Supplementary Material 9

Supplementary Material 10

## Data Availability

No datasets were generated or analysed during the current study.
