# Supplementary material for: Global dysregulation of circular RNAs in frontal cortex and whole blood from DM1 and DM2
Source: Hum Genet. 2025 Feb 4;144(4):417–32. doi: 10.1007/s00439-025-02729-x (PMC12003446; doi:10.1007/s00439-025-02729-x)
Supplement: Supplementary file 9 — Supplementary Material 9 [file 439_2025_2729_MOESM9_ESM.docx]

**SUPPLEMENTARY MATERIAL**

**Global Dysregulation of Circular RNAs in Frontal Cortex and Whole Blood**

**From DM1 and DM2**

Arvind Srinivasan1; Dorota Magner1,#, Piotr Kozłowski2, Anna Philips3, Arkadiusz Kajdasz3, Paweł Wojciechowski4,5, and Marzena Wojciechowska1,*

1, Department of Rare Diseases, Institute of Bioorganic Chemistry, Polish Academy of Sciences, Poznan, Poland; asrinivasan@ibch.poznan.pl

2, Department of Molecular Genetics, Institute of Bioorganic Chemistry, Polish Academy of Sciences, Poznan, Poland; kozlowp@ibch.poznan.pl

3, Department of Bioinformatics, Institute of Bioorganic Chemistry, Polish Academy of Sciences, Poznan, Poland; aphilips@ibch.poznan.pl; akajdasz@ibch.poznan.pl

4, Laboratory of Genomics, Institute of Bioorganic Chemistry, Polish Academy of Sciences, Poznan, Poland; Pawel.Wojciechowski@cs.put.poznan.pl

5, Institute of Computing Science, Poznan University of Technology, Poznan, Poland

#, Current address: Department of Biochemistry and Biotechnology, University of Life Sciences, Poznan, Poland; dorota.magner@up.poznan.pl

*, corresponding author, marzena.wojciechowska@ibch.poznan.pl

**Supplementary Figures: S1-S16**

**Supplementary Excel Tables: S1-S9**


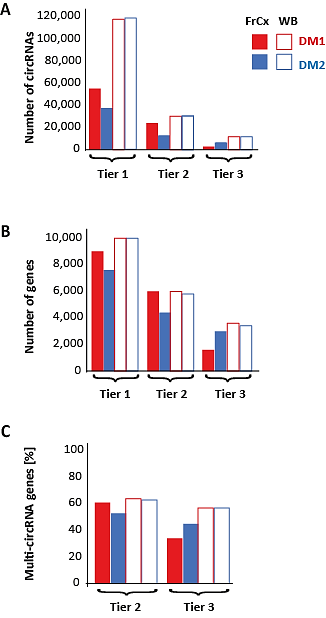


**Figure S1:** *The Number of CIRI2-identified CircRNAs and Their Genes.* (A) All circRNAs identified (Tier 1) and their further filtrations (Tier 2 and Tier 3); (B) Number of circRNA genes; (C) The fraction of genes giving rise to multiple circRNA species.


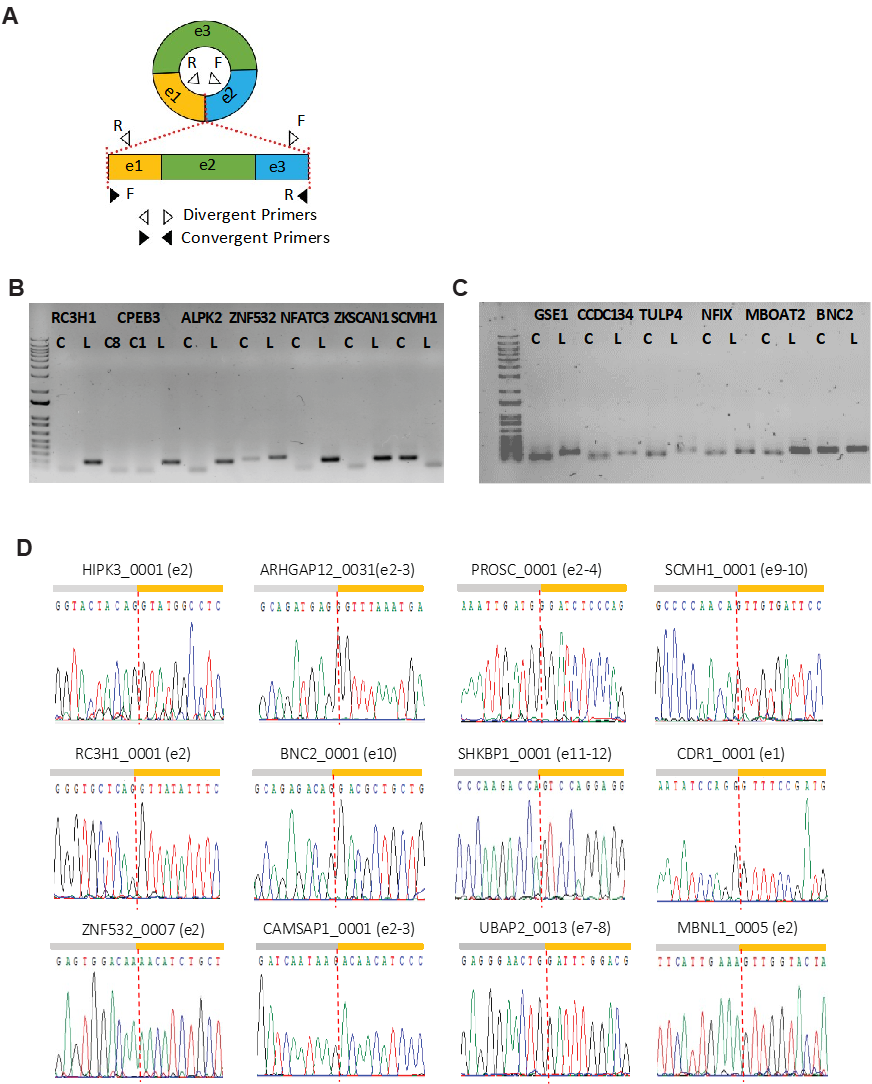


**Figure S2.** Continuation on the next page.


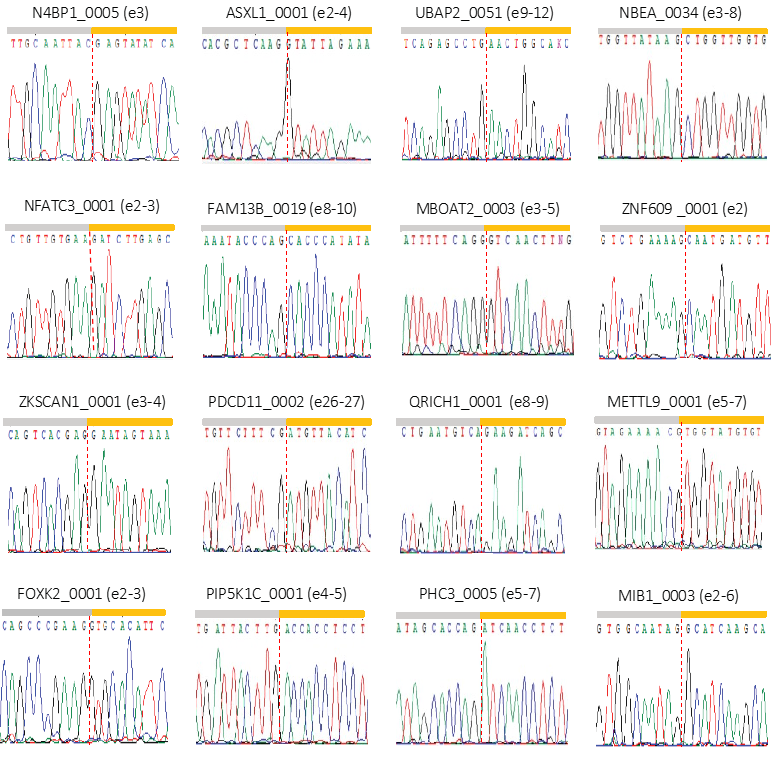


**Figure S2.** *CircRNA Validation Assays.* (A) Design of divergent and convergent primers for identification of, respectively, circRNAs and their linear host transcripts. (B, C) Representative agarose gels showing RT-PCR products of circRNAs (C) and linear mRNA (L). (D) In silico-identified nucleotide sequences at the back-splice junctions were validated via Sanger sequencing, and representative chromatograms are shown. All primers used are listed in Table S6. F, forward primer; R, reverse primer; e, exons; M, 1kb ladder; BSJ, back-splice junction.


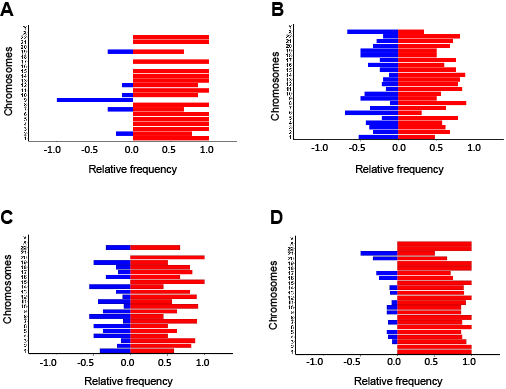


**Figure S3**. *Chromosomal Distribution of CircRNA Genes*. The relative frequency of circRNA genes from Tier 3 in human chromosomes is depicted. Red and blue bars indicate, respectively, upregulated and downregulated circRNAs from the FrCx of DM1 (A) and DM2 (B), as well as from the WB of DM1 (C) and DM2 (D). Positive and negative values of relative frequency indicate, respectively, upregulated and downregulated circRNAs across specific chromosomes, with the frequencies normalized to equal 1.


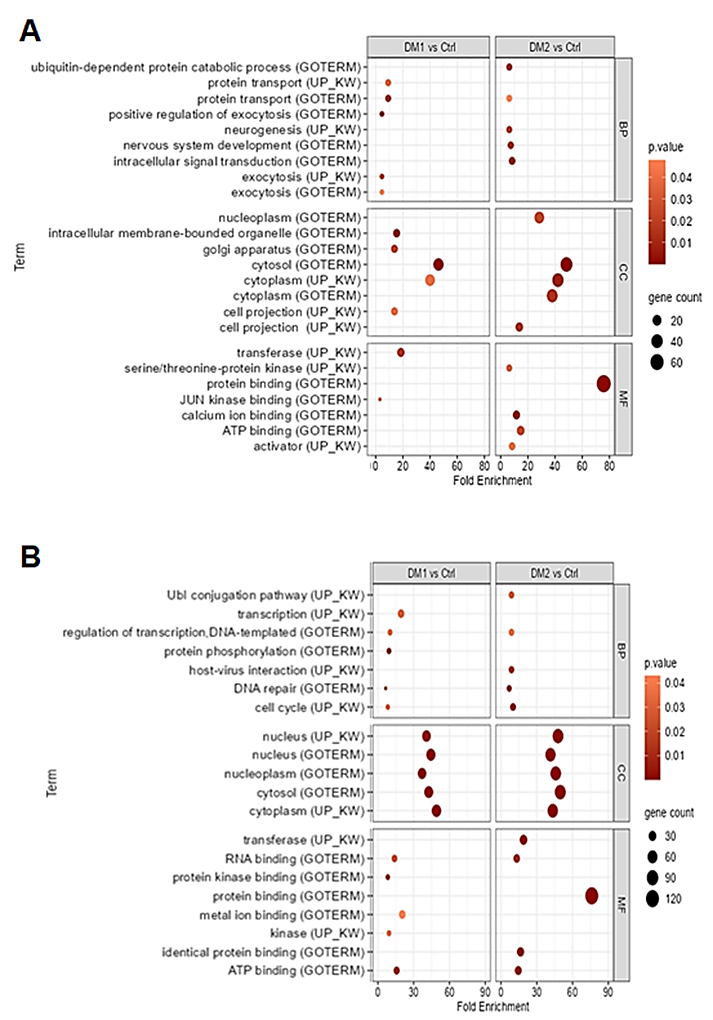


**Figure S4.** *Gene Ontology Analysis of CircRNA Genes*. The functional enrichment analysis of genes corresponding to upregulated circRNAs from the frontal cortex (A) and whole blood (B) was performed using DAVID Bioinformatics Resources (https://david.ncifcrf.gov/). Summary results for UniProt and Gene Ontology classified into three terms of biological process (BP), cellular component (CC) and molecular function (MF) are depicted.


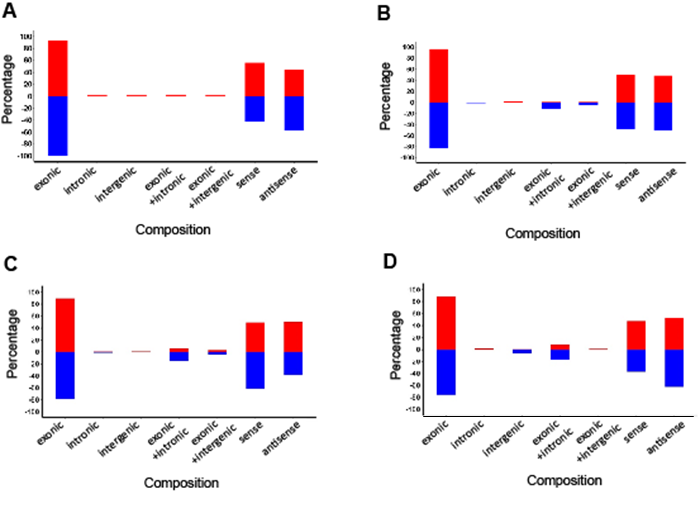


**Figure S5.** *Types and Counts of Differentially Expressed CircRNAs.* The CIRI2-identified circRNAs were classified into three major types (exonic, intronic, and intergenic) and mixed (exonic+intronic, exonic+intergenic) according to the relationship and genomic loci with their associated coding genes. Red and blue bars indicate, respectively, upregulated and downregulated circRNAs from the frontal cortex of DM1 (A) and DM2 (B), as well as from whole blood from DM1 (C) and DM2 (D). For each dataset of panels A-D, the total circRNA composition was normalized to 100%, with positive values indicating upregulation (red bars) and negative values indicating downregulation (blue bars).


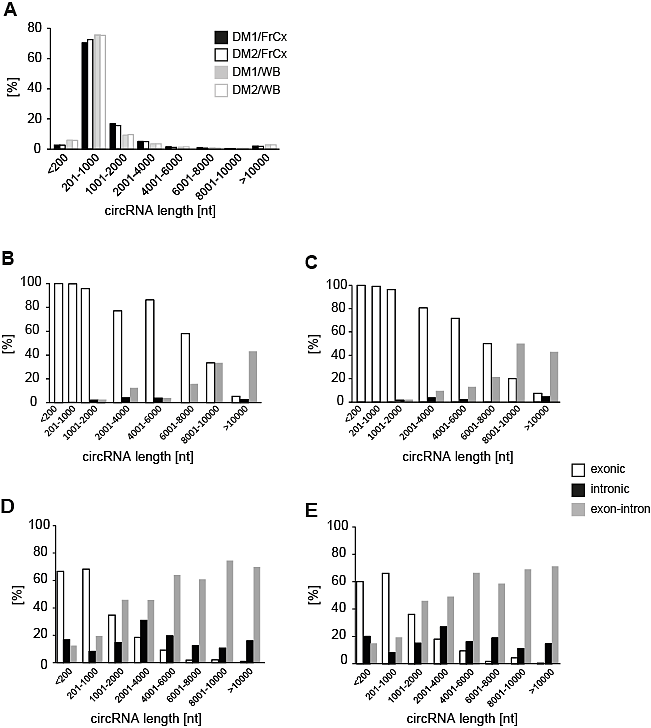


**Figure S6.** *CircRNA Composition and Length.* The plotted data are gathered in Excel Tables S2-S5 and are related to (A) the length of circRNAs (based on their coordinates) in three major categories, i.e., exonic, intronic, and exonic-intronic in the frontal cortex from DM1 (B) and DM2 (C), as well as in the whole blood from DM1 and DM2, respectively, (D) and (E).


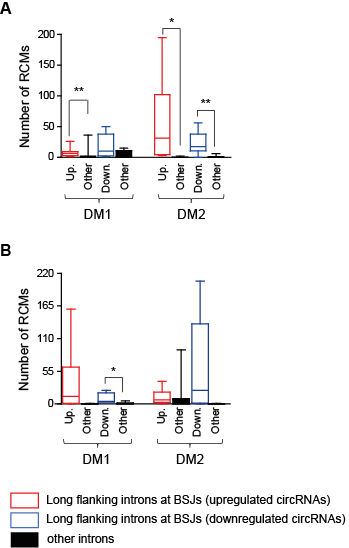


**Figure S7 (related to Figures 2A and 2B).** *The Number of Reverse Complementary Matches (RCMs) in Introns Flanking Exonic CircRNAs.* The number of RCMs in pairs of introns flanking circular BSJs (blue and red bars) and in other introns (black bars) in the frontal cortex (A) and the whole blood (B). Detailed data for this analysis are summarized in Table S7. Red and blue bars indicate, respectively, upregulated and downregulated circRNAs, whereas “other” indicates the RCMs between the other introns of circRNA genes away from BSJs. **p* < 0.05, ***p* < 0.001 (two-tail *t*-test).


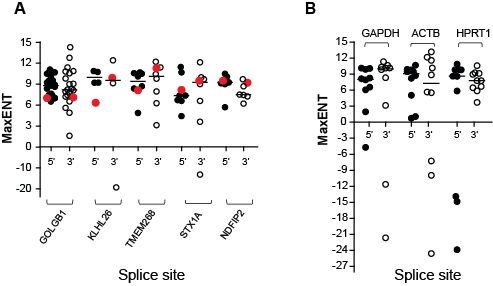


**Figure S8 (related to Figures 2C and 2D).** *The Strength of Splice Sites at the 5’ and the 3’ Ends of Circular and Linear Transcripts*. The maximum entropy (MaxEnt) score was used to measure the strengths of splice sites (“ss”) in individual circRNAs. (A) The MaxEnt scores calculated for all “ss” of depicted genes (red circles indicate the scores at the BSJs and black circles show the scores for other “ss” of the genes). (B) The MaxEnt values of all “ss” in three housekeeping genes devoid of circRNAs.


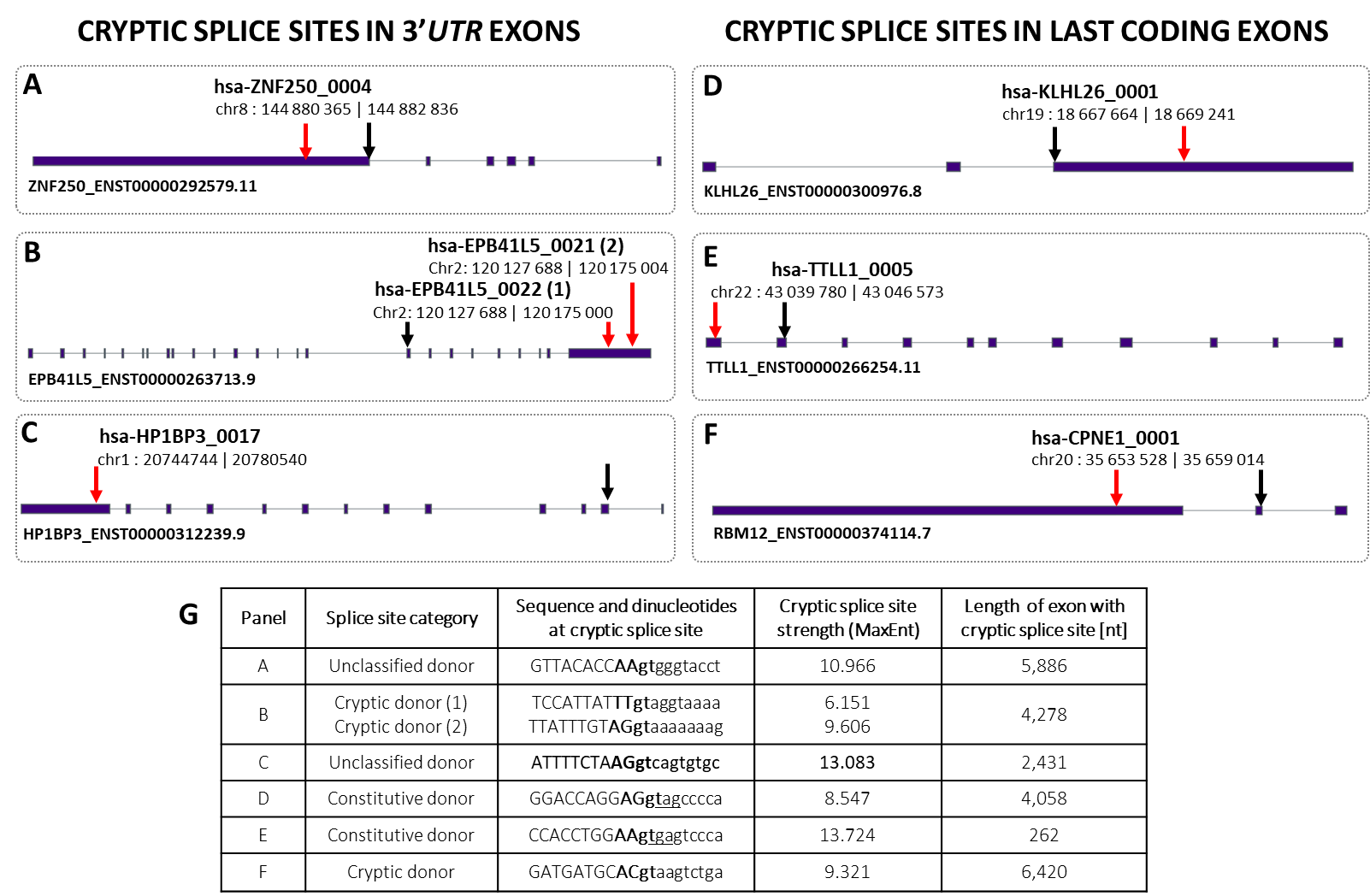


**Figure S9 (related to Table S8).** *Exonic Cryptic Splice Sites Engaged in BSJ Formation*. (A-C) The cryptic splice sites (“ss”) in the 3’-*UTR* exons of representative circRNAs. (D-F) The cryptic “ss” in the last coding exons of representative circRNAs. The schematic representation of the transcripts was downloaded from GTex Portal (https://www.gtexportal.org/home/); whereas the sequences of individual circRNAs were obtained from UCSC browser were used to determine the type of cryptic “ss” with the Alternative Splice Site Predictor (http://wangcomputing.com/assp/). (G) The summary of various features of cryptic splice sites. The complete summary data related to exonic cryptic splice sites in circRNAs from DM1 and DM2 are listed in Table S8. Red and black arrows indicate, respectively, the location of cryptic and canonical “ss”. CircRNA IDs and their coordinates are depicted in each panel.

**
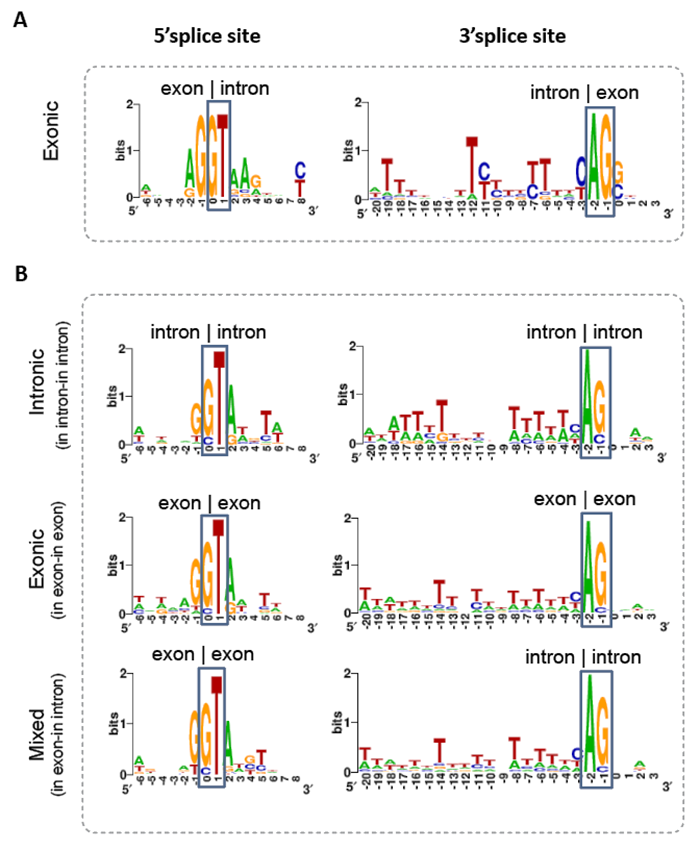
**

**Figure S10.** *WebLogo Analysis of Dinucleotides and Their Frequency at BSJs.*  CircRNAs with BSJs located in canonical and non-canonical positions were characterized according to: (A) both splice sites at canonical exon-intron boundaries; (B) cryptic splice sites in various locations i.e., both splice sites within intron(s) (upper panel); both splice sites within exon(s) (middle panel), and one splice site within exon and one within intron (lower panel).

**Figure S11**. *Expression Levels of CircRNAs With Canonical and Non-Canonical BSJs and Their Linear Host Transcripts.* Average RPMs of individual circRNAs from FrCx and WB for controls, DM1 and DM2 samples. All the data shown are related to Supplementary Tables 2-5. Orange labels, linear RNAs and blue labels, circRNAs.


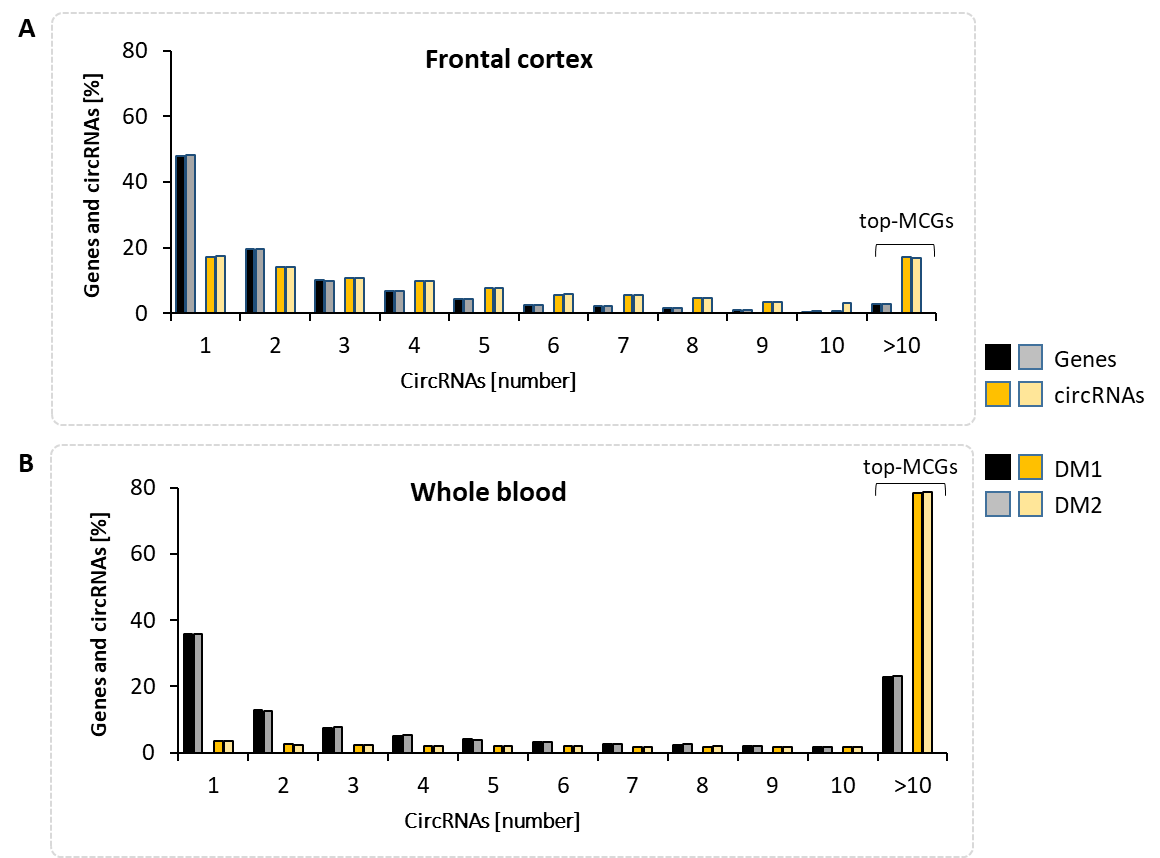


**Figure S12.** *CircRNAs Are Generated From Single-CircRNA Genes (SCGs) And Multi-CircRNA Genes (MCGs).* Bar graphs showing the percentage of genes that generate a particular number of distinct circRNA species in DM1 and DM2 (black and grey bars) and the percentage of circRNAs generated from these genes (orange and yellow bars) in FrCx (A) and WB (B). The top-MCGs hosting more than 10 distinct circRNAs constitute in the frontal cortex ~3% of all circRNA-generating genes, whereas in whole blood they represent ~23%.

**
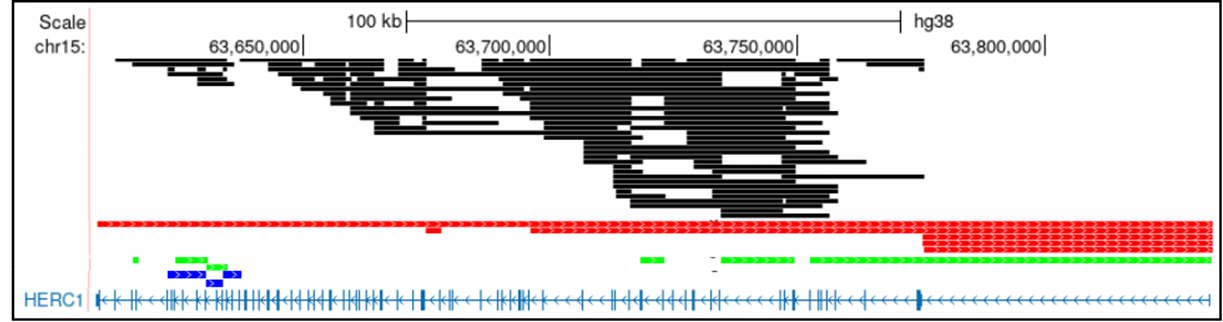
**

**Figure S13.**  *Schematic Illustration of HERC1 Representing Multi-CircRNA Gene from the Frontal Cortex*. The gene generates over 100 circRNAs (black bars) in FrCx, and merely 15 linear RNAs are listed in the Ensembl, and they include 6 protein-coding (red bars), 6 with intron retention (green bars), and 3 undergoing nonsense-mediated decay (blue bars). Data were illustrated with the UCSC Genome Browser.

**
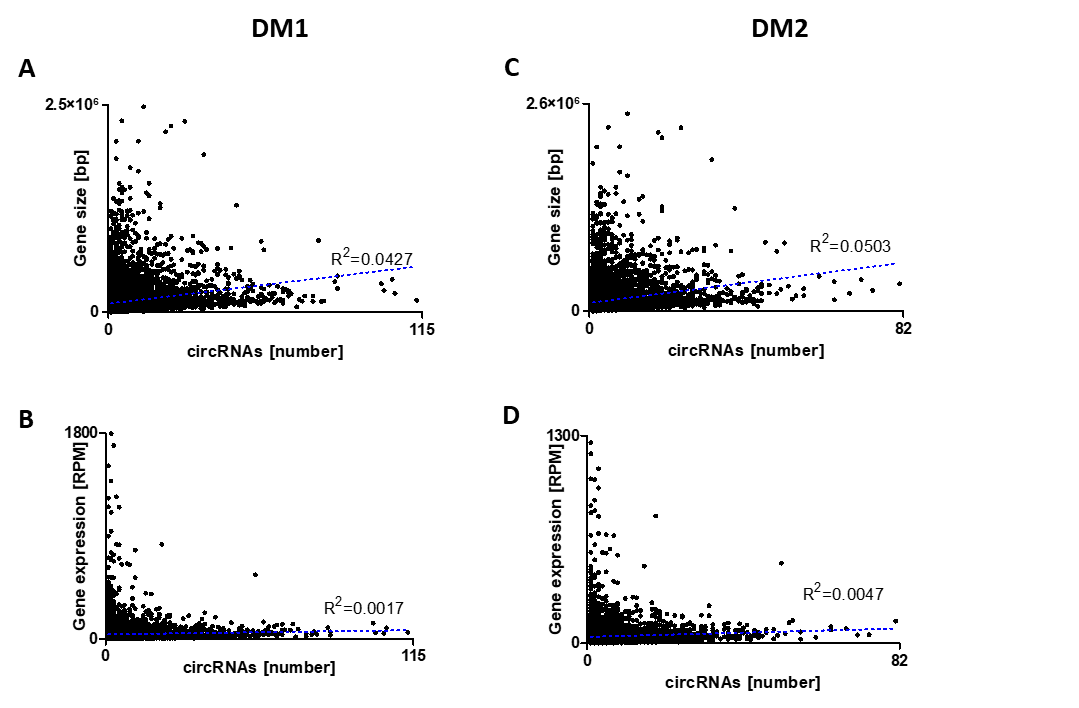
**

**Figure S14.** *The Correlation Between Genes Total Size and Their Expression Levels With CircRNA Abundance.* CircRNAs of SCGs and MCGs from the FrCx of DM1 and DM2 were used for the analysis. Gene expression data (B and D) were obtained from Otero BA et al., (2021). No association was found between these two qualities of circRNA genes and the number of circular species (A-D).

**
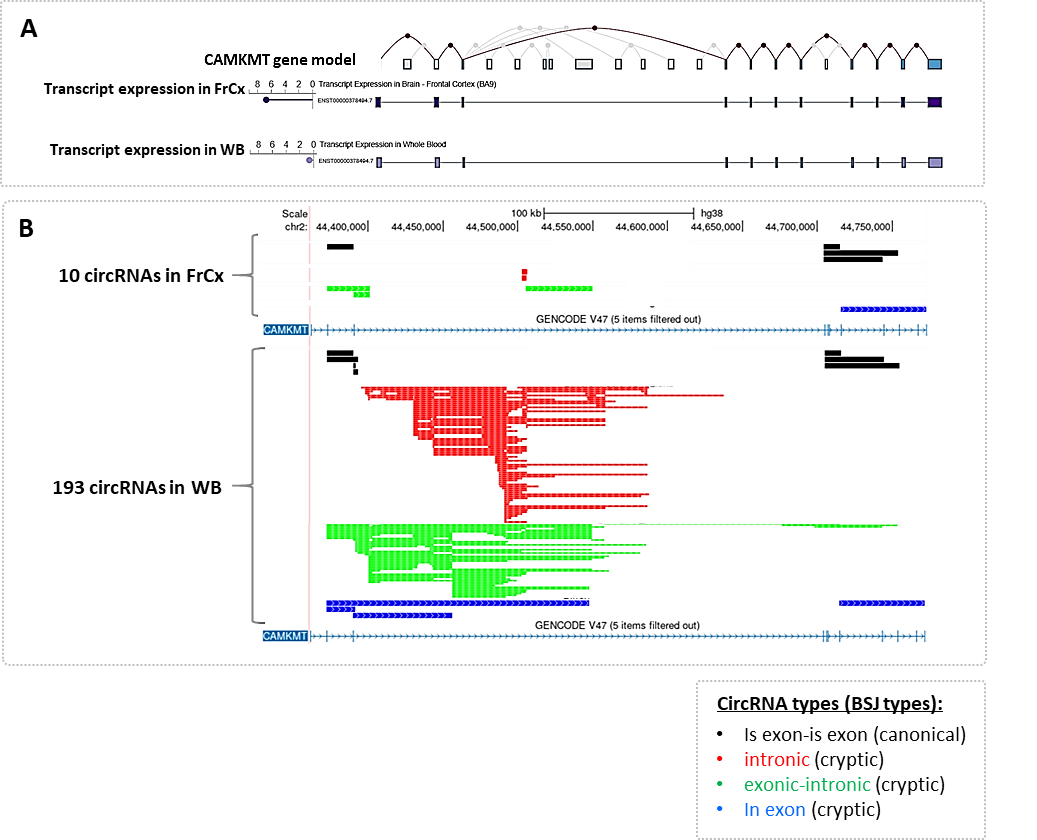
**

**Figure S15.** *Gene Expression Levels And the Quantity of CircRNAs*. A representative example of a multi-circRNA gene (*CAMKMT*), which could be transcribed into >190 circRNAs in the whole blood (WB) while only 10 are found in the frontal cortex (FrCx), regardless of its marginal expression in the blood. Based on transcriptomic data (Otero BA et al., 2021, and Sznajder L et al., 2020), expression of this gene in the FrCx or WB is not altered in DM1 and DM2 relative to controls. In panel (A), the gene model and its transcript levels in FrCx and WB are obtained from the GTEx Portal, while in panel (B), circRNA types (depicted in different colors of bars) and their distributions are illustrated with the UCSC genome browser.


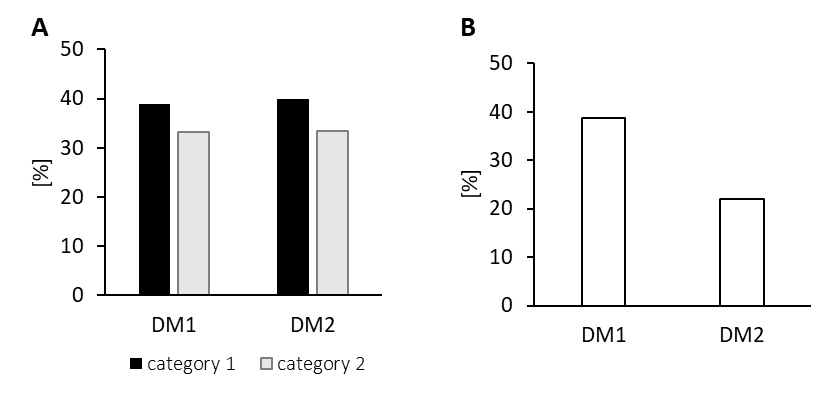


**Figure S16 (Related to Figure 5).** *CircRNA Isoforms and Alternative Splicing in Frontal Cortex from DM1 and DM2.*

(A) In DM1 and DM2 from the FrCx, almost 59% of all splicing events identified by Otero BA et al. (2021) were classified as cassette exons (CEs). These exons were found in different isoforms of circRNAs from CIRI2 output. Based on the CE presence/absence and the localization relative to BSJs, we identified two categories of circRNAs depicted in Figure 5 A-B. The category (1) is represented by hsa-PPHLN1_0001, hsa-PPHLN1_0002 and hsa-TRMT11_0005. These circRNA isoforms result from CE inclusion or exclusion, and in DM1 and DM2, they represent about 39% of all circRNAs (Table S2 and S3). The category (2) is represented by hsa-UBQLN1_0021 and hsa-SORBS1_0082 and indicates isoforms generated due to the differential occurrence of other alternative exons in circRNAs with consistent presence of the aberrantly spliced CE; this category represents 33% of all circRNAs identified in DM1 and DM2 from the FrCx (Table S2 and S3). These both categories feature isoforms with identical BSJs, thus the same circATLAS ID but with different lengths due to variations in body composition (Figure 5 A, B).

(B) The fraction of circRNA isoforms originated from the utilization of alternative 5’ or 3’ splice sites in DM1 and DM2 from the FrCx. Exemplary circRNAs of this kind are shown in Figure 5C and they include isoforms of KLHL24 and DGKE due to different 5’ donor splice sites and consistent acceptor splice sites in BSJs. Plotted is a fraction of circRNA isoforms (for genes giving rise to at least 4 circRNAs) that share one of the splice sites. These isoforms comprised nearly 39% and 22% of circRNAs identified from MCGs in DM1 and in DM2, respectively. The presence of such isoforms suggests the existence of hotspot regions driving circRNA formation.

**REFERENCES**

Otero, B.A., Poukalov, K., Hildebrandt, R.P., Thornton, C.A., Jinnai, K., Fujimura, H., Kimura, T., Hagerman, K.A., Sampson, J.B., Day, J.W., Wang, E.T. Transcriptome alterations in myotonic dystrophy frontal cortex. Cell Rep. 2021, 34(3):108634.

Sznajder, Ł.J., Scotti, M.M., Shin, J., Taylor, K., Ivankovic, F., Nutter, C.A., Aslam, F.N., Subramony, S.H., Ranum, L.P.W., Swanson, M.S. Loss of MBNL1 induces RNA misprocessing in the thymus and peripheral blood. Nat Commun. 2020, 11(1):2022.
